# Nutrition-First Support for GLP-1 and Dual Incretin Therapy in Obesity: A Practical Framework for Dietary Management, Symptom Tolerability, and Long-Term Weight Maintenance

**DOI:** 10.3390/nu18111751

**Published:** 2026-05-29

**Authors:** Raynier Zambrano-Villacres, Martín Campuzano-Donoso, Claudia Reytor-González, Gianluca Rossetti, Luigi Cobellis, Francesco Cobellis, Vincenzo Pilone, Daniel Simancas-Racines, Luigi Schiavo

**Affiliations:** 1 Ciencias de la Salud y Desarrollo Humano, Universidad ECOTEC, Km. 13.5, Samborondón 092302, Ecuador; razambrano@ecotec.edu.ec; 2 Facultad de Ciencias de la Salud y Bienestar Humano, Universidad Tecnológica Indoamérica, Ambato 180150, Ecuador; martincd01@hotmail.com (M.C.-D.);; 3 General and Bariatric Surgery Unit, Abano Terme Policlinic, 35031 Padova, Italy; gianlucarossetti@yahoo.it; 4 Unit of General Surgery, Casa Di Cura “Prof. Dott. Luigi Cobellis”, 84078 Vallo della Lucania, Italy; 5 Public Health Department, University of Naples Federico II, 80131 Naples, Italy; 6 Department of Medicine, Surgery and Dentistry “Scuola Medica Salernitana”, University of Salerno, 84081 Baronissi, Italy

**Keywords:** obesity, GLP-1 receptor agonist, dual incretin therapy, tirzepatide, semaglutide, nutrition therapy, protein intake, gastrointestinal tolerability, lean body mass, weight maintenance

## Abstract

Background: Glucagon-like peptide-1 receptor agonists (GLP-1RAs) and dual glucose-dependent insulinotropic polypeptide (GIP)/GLP-1 receptor agonists have transformed obesity treatment, producing substantial weight loss during active therapy. However, real-world effectiveness may be limited by gastrointestinal adverse events, reduced dietary intake, fat-free mass loss as part of total weight reduction, and weight regain after discontinuation. Methods: This narrative review synthesizes current pharmacological, nutritional, gastrointestinal, body-composition, and implementation evidence to propose an evidence-informed nutrition-first framework for patients receiving incretin-based therapy for obesity. Results: We translate pharmacologic mechanisms into practical dietary strategies, including protein prioritization, structured meal patterns, hydration and fiber management, symptom-targeted interventions, resistance-training support, and maintenance planning. Because direct trials of structured nutrition interventions in GLP-1RA- or dual incretin-treated populations remain limited, several recommendations are extrapolated from the broader obesity, caloric restriction, body-composition, gastrointestinal, and expert-consensus literature. Conclusions: Integrating structured nutrition care into pharmacotherapy pathways may help address meal-related symptom burden, support protein and fluid adequacy, identify patients at higher nutritional or body-composition risk, and prepare patients for long-term weight-management behaviors. Embedding practical nutrition management within multidisciplinary obesity care may help translate pharmacologic efficacy into durable, patient-centered outcomes.

## 1. Introduction

The therapeutic landscape of obesity management has changed profoundly with the clinical introduction and rapid uptake of glucagon-like peptide-1 (GLP-1) receptor agonists and dual glucose-dependent insulinotropic polypeptide (GIP)/GLP-1 receptor agonists (GLP-1RAs) [1,2]. These agents, including semaglutide and tirzepatide, have demonstrated levels of efficacy in weight reduction that were previously achievable primarily through metabolic and bariatric surgery [3,4,5]. Randomized clinical trials consistently report mean weight loss ranging from approximately 15% to more than 20% of total body weight, accompanied by improvements in glycemic control, cardiometabolic risk factors, and obesity-related comorbidities [6,7,8]. As a result, pharmacotherapy is increasingly positioned as an important component of contemporary obesity treatment algorithms rather than a secondary or last-line option [9]. However, practical guidance on how to integrate structured nutrition care with incretin-based pharmacotherapy remains limited, particularly regarding meal structure, gastrointestinal symptom management, protein adequacy, body-composition monitoring, and maintenance planning.

In this context, incretin-based pharmacotherapy should be viewed as complementary to, rather than a replacement for, established obesity interventions. Intensive lifestyle intervention remains the foundation of obesity care and typically produces mean weight reductions of approximately 5–8% of baseline body weight when delivered with structured dietary, physical-activity, and behavioral support [10,11,12,13]. Metabolic and bariatric surgery generally produces larger and more durable weight loss in eligible patients, with long-term outcomes varying by procedure, baseline characteristics, and follow-up duration [14,15]. GLP-1RAs and dual incretin therapies therefore occupy an important therapeutic space between lifestyle intervention and surgery: they can achieve substantial weight loss during active treatment, but their real-world impact depends on tolerability, persistence, nutritional adequacy, body-composition monitoring, and long-term maintenance planning [7,9,16].

Despite these advances, the translation of trial efficacy into sustained real-world effectiveness remains inconsistent [9,17,18]. In our previous comparative review examining incretin-based pharmacotherapy for insufficient weight loss and weight recurrence after metabolic and bariatric surgery, discontinuation and suboptimal long-term persistence emerged as key determinants of heterogeneous outcomes across clinical settings, despite overall favorable efficacy and safety profiles [17]. Discontinuation is driven by multiple factors. These include gastrointestinal (GI) adverse events, particularly nausea, vomiting, reflux, constipation, and early satiety, as well as financial barriers, insurance limitations, medication shortages, and patient uncertainty about long-term treatment expectations [19,20]. These barriers underscore the distinction between pharmacologic efficacy under controlled trial conditions and long-term therapeutic success in routine clinical practice [17].

Emerging real-world evidence indicates that a substantial proportion of individuals initiating GLP-1 receptor agonist therapy discontinue treatment within the first year [20]. Observational cohort analyses suggest that nearly half of patients stop therapy during this period, with discontinuation rates in individuals with obesity but without type 2 diabetes reported to be even higher in some populations [20]. In a large US cohort study of adults with overweight or obesity receiving dual-labeled GLP-1RAs, one-year discontinuation was higher among patients without type 2 diabetes than among those with type 2 diabetes, underscoring the importance of access, adherence, and long-term treatment planning in obesity pharmacotherapy [20]. Such patterns are clinically significant because obesity is now widely recognized as a chronic, relapsing disease requiring sustained intervention [21,22,23]. When pharmacotherapy is interrupted, appetite suppression and satiety enhancement diminish. This may be accompanied by increased hunger, reduced energy expenditure, and re-emergence of pre-treatment eating patterns, all of which can contribute to weight regain [24,25]. Clinical follow-up studies after medication discontinuation have demonstrated partial or substantial weight recidivism in many patients, reinforcing the need for long-term, integrated management strategies [26].

Within this context, nutrition care represents a clinically relevant but underdeveloped component of incretin-based obesity pharmacotherapy [27]. Current clinical guidance emphasizes medication selection, dose escalation, and safety monitoring but provides comparatively limited direction regarding structured dietary management during therapy [28]. By suppressing appetite, delaying gastric emptying, and reducing total energy intake, GLP-1 receptor agonists and dual incretin therapies can alter meal size, dietary variety, protein intake, hydration, fiber tolerance, and gastrointestinal symptom burden [17,29,30,31,32,33]. These effects support weight loss, but they may also create nutrition-management challenges when patients lack structured guidance on meal composition, portioning, hydration, symptom recognition, and body-composition risk [17,31,32,33].

Accordingly, this narrative review proposes a nutrition-first framework as a pragmatic, evidence-informed model for integrating nutrition support into incretin-based therapy. Rather than establishing formal guidelines, the framework organizes heterogeneous evidence into practical domains for baseline assessment, symptom-directed dietary counseling, body-composition risk monitoring, and maintenance planning [27,34].

This narrative review aims to synthesize current evidence and clinical practice insights to support a practical, nutrition-centered model of care for individuals receiving GLP-1 receptor agonists and dual incretin therapies for obesity. Rather than presenting new formal guidelines or validated nutrition protocols, this review advances the existing literature by offering a conceptual and translational framework that operationalizes incretin pharmacology into practical nutrition assessment domains, symptom-directed dietary strategies, body-composition risk monitoring, and maintenance-oriented clinical workflows. The focus is intentionally placed on dietary patterns, macronutrient distribution, and symptom-targeted nutrition strategies that can be implemented across clinical settings, rather than on micronutrient mapping alone. By integrating pharmacological mechanisms with applied nutrition management, this review seeks to provide clinicians with a pragmatic, evidence-informed guidance for supporting gastrointestinal tolerability, nutritional adequacy, body-composition monitoring, adherence-related behaviors, and long-term weight-maintenance planning.

## 2. Methodology

This article was designed as a narrative review with a structured literature search and evidence-informed synthesis. A narrative approach was selected because the objective was not to estimate pooled treatment effects. Instead, the aim was to integrate pharmacological, nutritional, body-composition, gastrointestinal, and implementation evidence into a clinically operational framework for nutrition support during GLP-1 receptor agonist and dual GIP/GLP-1 receptor agonist therapy for obesity. The heterogeneity of available evidence, including randomized pharmacotherapy trials, observational persistence studies, mechanistic studies, clinical nutrition trials, expert consensus documents, and practice-based reports, made a formal meta-analysis inappropriate for the aims of this review.

This narrative review was conducted through a structured literature search of PubMed, Scopus, and other relevant biomedical databases from inception through January 2026. Search terms were combined using Boolean operators and included “obesity,” “GLP-1 receptor agonist,” “GLP-1RA,” “dual incretin agonist,” “GIP,” “GIP/GLP-1,” “semaglutide,” “tirzepatide,” “gastrointestinal adverse events,” “nausea,” “vomiting,” “constipation,” “gastric emptying,” “lean body mass,” “fat-free mass,” “body composition,” “protein intake,” “leucine,” “resistance training,” “dietary patterns,” “Mediterranean diet,” “micronutrient,” “weight maintenance,” “weight regain,” “discontinuation,” and “persistence.” Reference lists of relevant clinical trials, reviews, consensus statements, and guidelines were also manually screened to identify additional sources.

Eligible sources included randomized controlled trials, extension and withdrawal trials, large observational studies, systematic reviews, meta-analyses, clinical practice guidelines, expert consensus statements, and mechanistic studies relevant to incretin-based pharmacotherapy, obesity nutrition care, gastrointestinal tolerability, body composition, dietary pattern quality, weight maintenance, and treatment discontinuation. Priority was given to evidence directly derived from GLP-1 receptor agonist or dual incretin-treated populations when available. Studies focused exclusively on diabetes management without relevant obesity, nutrition, gastrointestinal, body-composition, or weight-management outcomes were used only when mechanistically or clinically relevant. Narrative reviews and expert commentaries were used primarily for context or to identify clinical practice gaps, rather than as primary support for causal claims.

Because direct trials evaluating specific nutrition interventions during GLP-1 receptor agonist or dual incretin therapy remain limited, evidence was categorized according to its directness. Evidence was considered direct when derived from GLP-1 receptor agonist or dual GIP/GLP-1 receptor agonist trials, extension studies, withdrawal trials, or observational cohorts evaluating treated populations. Evidence was considered indirect when extrapolated from broader obesity, caloric restriction, body-composition, sarcopenia, resistance-training, bariatric, or gastrointestinal symptom-management literature. Expert consensus and practice-based guidance were considered separately and were used to inform pragmatic clinical considerations when direct empirical evidence was unavailable. Recommendations based primarily on indirect evidence or expert consensus are therefore presented as evidence-informed clinical considerations rather than formal graded guidelines.

When findings were discordant or evidence was limited, higher priority was given to randomized trials, large observational cohorts, systematic reviews, and meta-analyses, while mechanistic studies and expert consensus were used to contextualize clinical plausibility. Particular caution was applied when translating evidence from non-incretin weight-loss contexts to patients receiving GLP-1 receptor agonist or dual incretin therapy. These contexts include caloric restriction, bariatric surgery, sarcopenic obesity, and exercise nutrition. In these instances, the manuscript explicitly frames recommendations as extrapolated, hypothesis-generating, or practice-based. No formal risk-of-bias assessment, certainty grading, or quantitative synthesis was performed, consistent with the narrative design of the review.

## 3. From Pharmacology to Nutrition Priorities

Understanding nutrition priorities during incretin-based pharmacotherapy requires translating the pharmacodynamic actions of GLP-1RAs and dual GIP/GLP-1 agonists into concrete dietary implications [27]. Rather than revisiting pharmacology for its own sake, the clinical objective is to identify how these agents reshape appetite regulation, gastric motility, food preferences, and total energy intake, and how these changes should inform structured nutrition care [17].

GLP-1 is an incretin hormone secreted primarily from L-cells in the distal small intestine and colon in response to nutrient ingestion [35]. Pharmacologic GLP-1RAs reproduce and amplify its physiological actions, including glucose-dependent insulin secretion, suppression of glucagon, delayed gastric emptying, and central appetite regulation through hypothalamic and brainstem pathways [36,37,38]. These combined mechanisms drive substantial reductions in energy intake and body weight observed in randomized trials and real-world use [38]. A large-scale network meta-analysis of 76 randomized trials confirms these effects, demonstrating that while all GLP-1RAs improve glycemic control and weight management, combination therapies like dual-agonists like tirzepatide yield the most significant reductions in body weight (up to 14.03 kg) [39]. While these GI effects are therapeutic for weight loss, the associated slowing of gastric emptying and intestinal motility necessitates careful clinical management, particularly regarding prolonged fasting or prokinetic use, to mitigate risks such as retained gastric contents during anesthesia or endoscopic procedures [40]. The same mechanisms also alter meal patterns, eating behavior, and nutrient intake in ways that necessitate deliberate nutrition management to optimize tolerability and preserve nutritional adequacy [38].

### 3.1. Appetite Suppression and Early Satiety: Implications for Intake Structure

Central appetite modulation is a defining feature of incretin-based therapies. GLP-1 receptors located in the hypothalamus, brainstem, and reward-related regions of the central nervous system contribute to enhanced satiety, reduced hunger signaling, and diminished hedonic drive to eat [41,42]. Neuroimaging and behavioral studies demonstrate reduced activation of reward pathways in response to highly palatable foods during GLP-1 receptor agonist therapy, accompanied by decreased energy intake and altered food preferences [43]. A review on this topic presented that clinical trials consistently show substantial spontaneous caloric reduction, with average energy intake decreases ranging from approximately 15% to more than 30% depending on dose and population studied [44].

Patients frequently report a marked reduction in intrusive food-related thoughts and urges, a phenomenon colloquially described as the reduction of “food noise” [45]. While this effect facilitates adherence to energy restriction and supports weight loss, it also introduces a risk of insufficient protein, fiber, and fluid intake when meals are skipped or substantially reduced in size [46]. Early satiety and reduced appetite often lead to compressed eating windows and smaller portions, increasing the likelihood that patients fail to meet minimum intake thresholds necessary for lean mass preservation and metabolic stability [44,47].

From a nutrition management perspective, appetite suppression necessitates a shift toward structured intake prioritization [27]. Protein-forward meal construction, nutrient-dense food selection, and intentional meal timing become essential strategies to ensure adequate intake despite reduced hunger [27,48,49,50]. Rather than relying on intuitive eating cues alone, patients often benefit from a semi-structured eating pattern that ensures consistent protein and hydration intake across the day [51].

### 3.2. Delayed Gastric Emptying and Gastrointestinal Signaling

One of the primary mechanisms contributing to both weight loss and GI adverse events is delayed gastric emptying [40]. GLP-1RAs function in part as enterogastrones, slowing gastric motility and prolonging gastric distension following meals [29]. This prolongation enhances postprandial satiety but also increases the likelihood of nausea, fullness, reflux, and vomiting, particularly during dose escalation phases [40,52]. Clinical pharmacology studies confirm that GLP-1 receptor activation reduces gastric emptying rate and modulates vagal signaling, contributing to early satiety and reduced meal size [40].

The nutritional implications of pharmacologically slowed gastric emptying are substantial. Reduced gastric throughput effectively lowers functional meal capacity, meaning that larger or high-fat meals may provoke significant discomfort [40,53]. Observational and trial data consistently identify GI adverse events, especially nausea, vomiting, constipation, and reflux, as leading contributors to dose reduction or discontinuation [52]. A large-scale analysis involving 5442 cases further characterizes these GI profiles, noting that while severity may be influenced by age and body weight, the risk of these events typically follows an ‘early failure’ pattern that decreases over time as patients continue treatment [54]. A narrative review indicates that while GI side effects are the primary barrier to long-term therapy, these events are typically dose-dependent and manageable through individualized risk assessment and careful dose titration [19]. Many of these symptoms are highly sensitive to meal composition, portion size, and eating pace [55,56].

Accordingly, patients require explicit guidance on portioning logic and meal structure [55]. Smaller, more frequent meals; slower eating pace; careful titration of dietary fat; and attention to food texture can significantly improve tolerability [55,56]. Emphasis on nutrient density becomes critical because reduced gastric capacity limits total intake volume [57]. Ensuring that smaller meals deliver sufficient protein and overall dietary quality helps prevent unintended undernutrition while minimizing symptom burden [55].

### 3.3. Dual Agonism and the Role of GIP

Dual GIP/GLP-1RAs, such as tirzepatide, introduce additional metabolic effects that may influence nutritional priorities. In addition to GLP-1-mediated appetite suppression and delayed gastric emptying, GIP receptor activation appears to enhance adipocyte lipid handling, improve insulin sensitivity, and contribute to overall weight reduction through complementary mechanisms [58,59,60]. Data suggest that GIP signaling may support adipose tissue remodeling and metabolic flexibility, although its precise contribution to weight loss relative to GLP-1 remains an area of ongoing investigation [38,58].

Unlike GLP-1, GIP does not appear to exert a strong inhibitory effect on gastric emptying and may have neutral or modestly stimulatory effects on gastric motility in certain contexts [61]. A randomized crossover study in healthy volunteers distinguishes the incretin effects of these hormones, demonstrating that while both improve the insulinogenic index, GLP-1 specifically drives weight loss benefits through a unique combination of delayed gastric emptying and reduced hunger, effects that were not observed with GIP alone [61]. This distinction has led to hypotheses that dual agonists may demonstrate a somewhat different GI tolerability profile compared with high-dose GLP-1 monotherapy, although GI symptoms remain common across both classes [17,62]. For nutrition management, the central implication is that appetite suppression and reduced intake remain the dominant drivers of nutritional risk regardless of single or dual agonism [44].

From a nutrition-management perspective, the distinction between GLP-1RAs and dual GIP/GLP-1RAs is clinically relevant but should not be overstated. Dual incretin therapy may produce greater mean weight loss than many GLP-1 receptor agonist regimens, and GIP receptor activation may contribute additional effects on adipose tissue biology, insulin sensitivity, and metabolic regulation [63]. However, the practical nutrition priorities overlap substantially across both therapeutic classes because both can reduce appetite, lower total energy intake, alter meal size, and produce gastrointestinal symptoms during dose escalation. Therefore, nutrition care should be individualized according to the patient’s symptom profile, rate of weight loss, intake adequacy, body-composition risk, comorbidities, and treatment phase rather than assuming that GLP-1RAs and dual incretin therapies require entirely separate dietary algorithms [19,40,63].

### 3.4. Energy Intake Reduction and Body Composition Considerations

Substantial reductions in spontaneous energy intake are consistently observed during incretin therapy [64]. Controlled feeding and free-living studies demonstrate decreases in caloric intake ranging from approximately 16% to nearly 40%, accompanied by shifts away from high-fat and highly processed foods [27,44]. While this energy reduction underpins weight loss efficacy, it also raises concerns regarding preservation of lean body mass and overall dietary adequacy [65]. Rapid or profound caloric reduction without structured protein intake and resistance training may predispose patients to disproportionate loss of fat-free mass, particularly in older adults or individuals with low baseline muscle reserves [27]. Randomized trial data in older adults with obesity demonstrate that resistance training maintains muscle strength and physical function during caloric restriction-induced weight loss, even when some lean mass is reduced, supporting the combined use of resistance exercise and adequate protein intake to mitigate sarcopenic risk [66].

Evidence from obesity and bariatric populations indicates that preservation of lean mass during weight loss requires sufficient protein intake distributed across meals and supported by resistance-based physical activity [67,68,69]. Without deliberate nutrition planning, medication-induced appetite suppression can lead to protein intakes below recommended thresholds, especially when patients consume only one or two small meals per day [61]. Fatigue, reduced physical activity, and diminished functional capacity may follow if energy and protein intake fall excessively [68].

### 3.5. Translating Pharmacology into Practical Nutrition Priorities

Taken together, the pharmacological effects of GLP-1 receptor agonists and dual incretin therapies create a focused set of nutrition-management challenges: reduced appetite, lower meal capacity, altered food preferences, gastrointestinal symptoms, and potential inadequacy of protein, fluid, fiber, and micronutrient intake in susceptible patients [38,47,61]. These priorities are evidence-informed and mechanistically grounded, but direct trials demonstrating that specific nutrition strategies improve medication persistence, reduce adverse-event rates, or preserve fat-free mass during incretin therapy remain limited [27].

**Figure 1:**
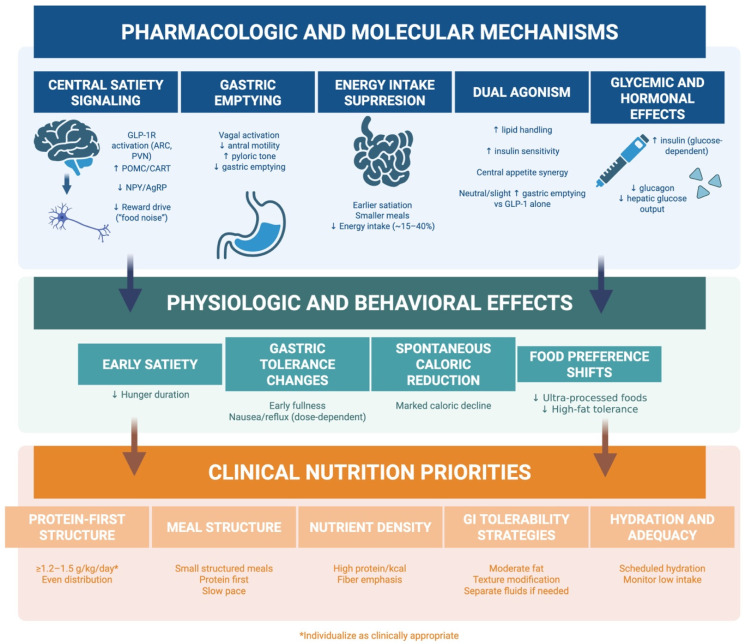
Mechanism-driven nutrition priorities during GLP-1 receptor agonist and dual incretin therapy for obesity. Glucagon-like peptide-1 receptor agonists (GLP-1RAs) and dual glucose-dependent insulinotropic polypeptide (GIP)/GLP-1RAs produce weight loss through integrated central and peripheral mechanisms, including hypothalamic satiety signaling, modulation of mesolimbic reward pathways, delayed gastric emptying via vagal pathways, and substantial reductions in spontaneous energy intake. These pharmacologic effects lead to predictable physiologic and behavioral changes, such as early satiety, reduced meal size, gastrointestinal symptoms, and decreased caloric intake, that directly shape dietary patterns and nutritional risk. Translating these mechanisms into structured nutrition priorities may help clinicians identify patients at risk of inadequate intake, gastrointestinal intolerance, and excessive fat-free mass loss, while guiding individualized considerations for protein adequacy, hydration, fiber titration, meal structure, and symptom monitoring. The proposed priorities integrate direct evidence on incretin pharmacology and adverse-event profiles with indirect evidence from broader clinical nutrition, body-composition, and gastrointestinal symptom-management literature [27,29,38,40,47,53,55,56,64,70,71,72,73]. Abbreviations: GLP-1, glucagon-like peptide-1; GLP-1R, glucagon-like peptide-1 receptor; ARC, arcuate nucleus; PVN, paraventricular nucleus; POMC, pro-opiomelanocortin; CART, cocaine- and amphetamine-regulated transcript; NPY, neuropeptide Y; AgRP, agouti-related peptide; ↑ increase/enhancement; ↓ decrease/reduction.

Key priorities include protein-forward meal structuring, attention to nutrient density, gradual fiber titration to support GI function, and proactive symptom management through portion control and meal timing strategies. By aligning nutrition care with pharmacologic mechanisms, clinicians may help patients anticipate reduced meal capacity, maintain adequate protein and fluid intake, and apply symptom-targeted dietary adjustments during dose escalation and active weight loss. Although these strategies are clinically plausible and supported by mechanistic rationale, expert consensus, and indirect evidence from the broader weight-loss and gastrointestinal literature, direct trials demonstrating improved persistence, reduced adverse-event rates, or superior body-composition outcomes during incretin therapy remain limited. The translation of incretin pharmacology into practical nutrition priorities is summarized in Figure 1, highlighting the mechanistic pathways through which these agents alter appetite, gastric motility, and dietary intake and the corresponding individualized nutrition considerations that may support tolerability, nutritional adequacy and long-term monitoring.

## 4. Pre-Treatment Assessment and Readiness

### 4.1. Setting the Nutritional Foundation Prior to Pharmacotherapy

Before initiating GLP-1 receptor agonist or dual incretin therapy, a focused pre-treatment nutrition assessment is essential to identify baseline intake patterns that may influence tolerability, adherence, and body-composition outcomes during treatment [27,74,75]. Real-world evidence indicates that GI adverse effects, low dietary quality, and inadequate protein or fluid intake contribute to early discontinuation and suboptimal outcomes, underscoring the need to evaluate nutritional readiness before dose escalation begins [76]. Baseline assessment should therefore identify patients at risk for inadequate protein, fiber, fluid, and micronutrient intake during dose escalation [77,78]. This assessment is intended to guide anticipatory counseling and monitoring rather than to predict tolerability with certainty.

For routine clinical translation, pre-treatment assessment can be supported by brief, validated tools and simple checklists rather than lengthy dietary evaluations in all patients. Depending on local workflow and clinical resources, clinicians may combine a structured diet history with a brief diet-quality screener, such as the Rapid Eating Assessment for Participants–Short Version (REAP-S) or a Mediterranean diet adherence screener; a protein-intake checklist; a bowel-pattern assessment using stool frequency and stool form; a GI symptom checklist or validated symptom scale; a hydration checklist; and validated eating disorder screening tools such as the SCOFF questionnaire when clinically indicated [79,80,81,82]. These tools should support risk identification, counseling, and monitoring, rather than function as rigid eligibility criteria for pharmacotherapy.

### 4.2. Baseline Diet Quality and Meal Structure

Most individuals presenting for obesity pharmacotherapy demonstrate dietary patterns characterized by high energy density and low overall diet quality, often with irregular meal timing and reliance on ultra-processed foods [83]. Observational studies in obesity populations consistently show that poorer baseline diet quality and erratic meal patterns are associated with reduced adherence to weight-loss interventions and less favorable body-composition changes during caloric restriction. A secondary analysis of a large, randomized weight-loss trial in generally healthy adults demonstrated that participants who improved both diet quality and adherence to macronutrient targets achieved significantly greater reductions in BMI over 12 months than those with poorer diet quality and adherence, highlighting the combined importance of dietary quality and consistency for successful weight loss [73]. Similarly, longitudinal cohort data from the MRC National Survey of Health and Development showed that healthier dietary pattern scores across midlife were associated with lower fat mass and more favorable adiposity distribution in later adulthood, supporting the role of sustained diet quality in shaping long-term body composition [84]. Assessing baseline dietary pattern, whether through brief validated tools such as Mediterranean diet adherence scores or through structured diet history, can help identify patients whose intake is dominated by energy-dense, low-protein foods that may be poorly tolerated once appetite declines [72,85,86]. Particular attention should be paid to meal timing and frequency, as prolonged fasting periods followed by large meals can exacerbate nausea, reflux, and early satiety when gastric emptying is pharmacologically delayed [27,87,88,89]. Establishing a predictable meal structure prior to treatment initiation improves tolerance and supports more consistent nutrient intake once appetite suppression occurs [33,71].

Protein, fluid, and fiber intake patterns warrant specific assessment given their central role in maintaining nutritional adequacy and GI function during incretin therapy [78,90]. Clinical studies demonstrate that GLP-1-based treatments significantly reduce spontaneous energy intake, which can inadvertently lower protein consumption below levels required to preserve lean body mass during weight loss [91]. Inadequate protein intake during energy restriction has been associated with greater fat-free mass loss and functional decline, particularly in older adults and individuals with sarcopenic obesity [92]. In a 20-week randomized weight-loss trial in postmenopausal women aged 50–70 years, higher dietary protein intake during caloric restriction was significantly associated with reduced total and appendicular lean mass loss, even after adjustment for intervention group and body size, supporting the importance of adequate protein intake to mitigate adverse body-composition changes during weight reduction [93]. Similarly, insufficient fluid intake is a common but underrecognized contributor to treatment-related nausea, fatigue, and constipation, while low baseline fiber intake increases the likelihood of constipation once gastric emptying slows and food volume decreases [56]. Expert consensus on nutrition management for GLP-1 therapy emphasizes the importance of adequate macronutrient and fluid intake alongside resistance training to preserve muscle and mitigate adverse effects such as nausea and dehydration-related complications [70]. Evaluating typical daily protein intake and distribution, hydration habits, and fiber exposure before medication initiation may support individualized counseling on protein prioritization, scheduled fluid intake, and gradual fiber titration, while recognizing that direct evidence linking these pre-treatment adjustments to reduced intolerance remains limited.

Micronutrient adequacy should also be considered during baseline assessment and follow-up, particularly because appetite suppression, early satiety, reduced dietary variety, and persistent gastrointestinal symptoms may reduce overall micronutrient intake during therapy [94]. Current evidence does not support universal micronutrient supplementation for all patients receiving GLP-1 receptor agonist or dual incretin therapy; however, individualized monitoring may be appropriate in patients with low baseline dietary quality, prolonged nausea or vomiting, diarrhea, restrictive eating patterns, older age, prior metabolic or bariatric surgery, chronic kidney disease, or other conditions associated with nutritional vulnerability. When clinically indicated, assessment may include dietary review and targeted laboratory evaluation of nutrients such as vitamin D, vitamin B12, iron status, folate, calcium, magnesium, and electrolytes, with supplementation guided by documented deficiency, inadequate intake, or established clinical indication rather than routine use [28,95].

### 4.3. Constipation Risk, Gastrointestinal History, and Meal Timing Patterns

Pre-existing GI symptoms are clinically relevant for anticipatory counseling during GLP-1RA or dual incretin therapy. Patients with baseline constipation, reflux, irritable bowel symptoms, rapid eating patterns, large evening meals, or high dietary fat intake may be more likely to experience symptom exacerbation during dose escalation [56]. GI adverse events are consistently reported as common reasons for dose adjustment, delayed escalation, or discontinuation in clinical and real-world settings [20,96,97]. Screening for bowel habits, stool frequency, habitual fat intake, eating speed, and typical meal size provides clinically actionable information [98,99]. Patients who routinely consume large evening meals or eat rapidly may benefit from anticipatory counseling on portion reduction, slower eating pace, and earlier meal timing to mitigate nausea and reflux during dose escalation [98,100,101,102]. Addressing constipation risk through hydration optimization, gradual fiber introduction, and physical activity recommendations prior to treatment initiation could improve early tolerability.

### 4.4. Screening for Restrictive or Disordered Eating Risk

Although incretin therapies are being investigated for their potential effects on binge-eating symptoms, their potent appetite-suppressing effects may also mask or exacerbate restrictive eating patterns in vulnerable patients [103]. A recent systematic review of GLP-1RAs in binge eating disorder and bulimia nervosa reported reductions in binge-eating frequency and related psychopathology in small pilot studies, while emphasizing the need for larger randomized trials to establish efficacy and safety [104]. Therefore, GLP-1RAs and dual incretin therapies should not be presented as established treatments for eating disorders, and appetite suppression should not be equated with recovery, normalization of eating behavior, or adequate nutritional intake [104,105].

Screening for a history of disordered eating, including binge eating, chronic restrictive dieting, or significant weight suppression, is therefore appropriate when clinically indicated [106,107]. The purpose of screening is risk identification and care planning, not automatic exclusion from obesity pharmacotherapy. Brief validated tools such as the SCOFF questionnaire or Eating Disorder Diagnostic Scale can be incorporated into pre-treatment evaluation to identify patients who may require closer monitoring or multidisciplinary support [108,109,110]. In addition to brief screening instruments, multidimensional progress-monitoring tools have been developed and validated to track eating disorder symptoms and related behavioral and psychological domains over time, supporting structured identification and follow-up of patients at higher risk [111]. Importantly, rapid reductions in appetite and food preoccupation during therapy may be perceived as therapeutic success, even when total intake becomes nutritionally inadequate [27,44]. Patients with active eating disorder symptoms, significant restrictive tendencies, recurrent binge eating, purging behaviors, or severe weight/shape concerns should be managed with multidisciplinary oversight, ideally involving obesity medicine, nutrition, and mental health professionals with eating disorder expertise [103,105,106]. Structured guidance on minimum intake targets, protein prioritization, regular meal patterns, and symptom monitoring may help ensure that pharmacotherapy supports nutritional adequacy and sustainable weight management without reinforcing maladaptive restriction.

For pragmatic implementation, patients may be categorized into lower or higher nutritional risk before treatment initiation. Lower-risk patients may have regular meal patterns, adequate baseline protein and fluid intake, minimal GI symptoms, preserved muscle strength, no major restrictive or binge-eating risk, and reliable follow-up access. Higher-risk features include low baseline protein intake, poor diet quality, chronic constipation or reflux, low fluid intake, older age, sarcopenic obesity or low muscle strength, prior metabolic or bariatric surgery, chronic kidney disease, restrictive or disordered eating risk, persistent vomiting or diarrhea, and anticipated barriers to follow-up. Patients with higher nutritional risk may benefit from earlier dietitian involvement, closer monitoring during dose escalation, and more individualized protein, hydration, fiber, micronutrient, symptom-management, and physical-activity planning. A structured framework for pre-treatment nutrition assessment and targeted clinical action is summarized in Table 1.

**Figure 2:**
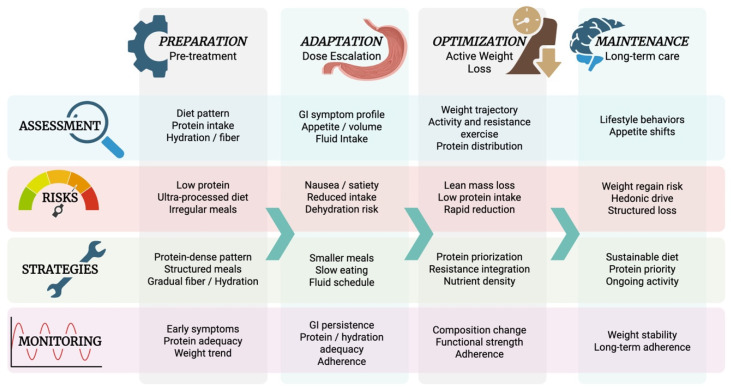
Evidence-informed nutrition support across phases of incretin therapy. This schematic illustrates how nutrition support may be integrated across the major phases of incretin-based therapy, from pre-treatment assessment through dose escalation, active weight loss, and long-term maintenance. Across phases, the framework highlights key assessment domains, common nutrition-related risks, and evidence-informed areas of focus relevant to tolerability, body composition, and sustainability. Cross-phase monitoring of symptoms, intake adequacy, treatment persistence, and weight trajectory may help contextualize evolving nutrition needs during treatment [22,27,32,44,56,77,78,120].

While Table 1 summarizes key domains for pre-treatment nutrition assessment, nutrition support during incretin therapy extends beyond the initial evaluation phase. As treatment progresses through dose escalation, active weight loss, and long-term maintenance, different nutrition-related risks and priorities emerge. To contextualize these phases within a clinical workflow, Figure 2 illustrates an operational framework for integrating nutrition assessment, risk identification, evidence-informed strategies, and ongoing monitoring throughout the course of incretin-based therapy.

## 5. Symptom-Targeted Nutrition Considerations to Improve Tolerability

### 5.1. The Clinical Importance of Symptom-Directed Nutrition Management

GI adverse events are the most frequently reported side effects of GLP-1RAs and dual incretin therapies and are clinically relevant contributors to dose-escalation failure and early treatment discontinuation [26]. Across major obesity trials, nausea, vomiting, diarrhea, and constipation are commonly reported, with nausea often affecting approximately 20–40% of treated participants depending on agent, dose, population, and ascertainment method [6,7,16,121]. These symptoms are typically most pronounced during dose escalation and are dose-dependent [6,19]. Because many symptoms are meal-related, dietary and behavioral strategies may help reduce symptom burden in selected patients, although direct evidence that these strategies improve long-term adherence remains limited [32,101]. Accordingly, the approaches in this section should be interpreted as pragmatic, individualized clinical considerations rather than validated symptom-management algorithms.

In practice, symptom-directed nutrition management can be approached stepwise. First, clinicians should assess symptom severity, hydration status, oral intake, bowel pattern, recent dose escalation, and alarm features such as persistent vomiting, severe abdominal pain, progressive distension, dehydration, or inability to maintain intake. Second, meal pattern and exposure to common triggers should be reviewed, including large portions, rapid eating, high-fat meals, late evening meals, carbonated beverages, alcohol, sugar alcohols, low fluid intake, and abrupt fiber increases. Third, first-line dietary adjustments should focus on smaller structured meals, slower eating pace, protein prioritization in tolerable portions, moderation of dietary fat, scheduled fluid intake between meals, and gradual fiber titration according to symptoms. Fourth, symptom-specific strategies can then be applied for nausea, reflux, constipation, diarrhea, or bloating, as summarized in Table 2. Finally, persistent, severe, or progressive symptoms should prompt medical reassessment, consideration of dose-escalation delay or dose adjustment, and evaluation for alternative diagnoses or complications rather than relying on dietary modification alone [40,56,121].

Brief patient-centered counseling messages may improve translation of symptom-targeted strategies into daily practice. Clinicians can explain early satiety by advising patients that “fullness may arrive earlier than expected, so the goal is to stop at comfortable fullness rather than finish the plate.” Portion control can be framed as “start with a smaller portion, eat slowly, and pause midway before deciding whether more food is needed.” Eating pace can be addressed by encouraging patients to take smaller bites, chew thoroughly, and avoid eating while distracted. For hydration, patients may be advised to sip fluids between meals rather than drinking large volumes with meals if fullness or reflux occurs. For constipation or bloating, fiber should be increased gradually rather than abruptly. These messages should be individualized according to symptom severity, cultural food practices, food access, comorbidities, and eating disorder risk [40,56]. These counseling points are intended as practical communication tools and should not be interpreted as evidence that dietary counseling alone prevents gastrointestinal adverse events or medication discontinuation.

Because randomized trials have not yet established symptom-directed dietary algorithms specifically for incretin-treated populations, the strategies discussed below should be interpreted as pragmatic, individualized clinical considerations. Their purpose is to help clinicians identify modifiable meal-related triggers, maintain hydration and nutrient intake, and determine when symptoms require medical reassessment rather than dietary adjustment alone.

### 5.2. Nausea and Vomiting

Nausea is the most common adverse event during incretin therapy and is strongly associated with delayed gastric emptying and heightened gastric distension signaling [40]. Symptoms are often most severe in the first days following injection or after dose increases [19]. In the STEP randomized trials of semaglutide for obesity, nausea and vomiting were the most frequently reported gastrointestinal adverse events and were typically transient, mild-to-moderate in severity, and occurred most often during treatment initiation and dose escalation [16,121,122]. Continuing to eat beyond the first sensation of fullness can precipitate nausea and vomiting due to reduced gastric clearance and prolonged gastric retention [123].

Nutritional management should focus on volume control, pacing, and food composition. Patients benefit from explicit guidance to stop eating at early satiety cues and to prioritize smaller, structured meals [27,72]. Lower-fat meals are generally better tolerated, as dietary fat further slows gastric emptying and may exacerbate symptoms [124]. During symptomatic periods, lower-fat, more easily digested carbohydrate and meal compositions may be better tolerated than high-fat, heavily seasoned foods, as clinical guidance suggests modifying dietary fat and food consistency to minimize nausea and improve gastrointestinal tolerability during GLP-1 therapy [27,125]. Adequate hydration is also critical, as reduced fluid intake during appetite suppression may exacerbate nausea, fatigue, constipation, and in vulnerable patients may contribute to dehydration-related renal complications [126,127]. Small, frequent sips of fluids between meals are often better tolerated [56]. Temperature and sensory modifications may further reduce symptoms, as cold or room-temperature foods are frequently perceived as less nauseating than hot, aromatic dishes [27,56].

### 5.3. Constipation

Constipation is common during GLP-1-based therapy and may persist for longer than nausea [16,40]. Slowed gastric emptying and reduced overall food intake decrease luminal bulk, while reduced fluid intake further impairs stool transit [40,128]. Additionally, the marked reduction in total food intake commonly observed during incretin therapy may further reduce stool bulk and intestinal motility, contributing to constipation if fiber and fluid intake are not intentionally maintained. Observational data and trial reports consistently identify constipation as a frequent reason for patient discomfort and dose adjustment. In the STEP pooled analysis of phase III semaglutide trials, constipation was among the most frequently reported gastrointestinal adverse events alongside nausea and vomiting, highlighting its clinical significance during therapy [121]. Similarly, in the STEP 5 semaglutide randomized trial, constipation was consistently observed in the semaglutide treatment arm as a common adverse event, supporting the need for proactive symptom management [129]. 

Data indicate that proactive management may be better than a reactive approach. Gradual fiber titration is preferred over abrupt increases, as rapid escalation can exacerbate bloating and abdominal discomfort [98]. Bellini et al. [98] recommend that enrichment of the diet with fiber should be slow and gradual to avoid or reduce disturbances such as bloating, flatulence, and intestinal cramps, particularly in the context of constipation management. A systematic review and meta-analysis of randomized controlled trials found that, while fiber improves stool frequency and consistency, higher doses (e.g., >10 g/day) and longer treatment durations are necessary for optimal effect, and adverse effects such as flatulence and bloating are more common with higher fiber doses [130]. A total fiber intake target consistent with general dietary evidence-informed clinical considerations (approximately 25–35 g/day, individualized to tolerance) is reasonable, though baseline intake should guide progression [131]. Soluble fiber sources, including oats, apples, and psyllium, may improve stool consistency and promote transit. Psyllium is particularly useful due to its gel-forming properties and minimal fermentation compared with some other fibers, which may reduce bloating while normalizing stool form [132]. Adequate hydration and regular physical activity should be emphasized alongside fiber adjustments to support bowel motility [130,131]. In patients with persistent constipation despite adequate hydration and gradual fiber titration, magnesium-based osmotic agents may be considered as adjunctive therapy to improve stool water content and intestinal motility [133].

### 5.4. Diarrhea and Bloating

Although less common than constipation, diarrhea and bloating can occur, particularly during early treatment or rapid dose escalation. These symptoms may reflect alterations in intestinal transit, changes in bile acid handling, or increased sensitivity to dietary fat [19,134]. High-fat meals, spicy foods, sugar alcohols, and excessive caffeine may exacerbate gastrointestinal symptoms in susceptible individuals, as these dietary factors are known to worsen dyspepsia, bloating, and diarrhea through effects on gastric motility, visceral sensitivity, and osmotic load [27,135,136]. Nutritional strategies focus on reducing luminal irritants and stabilizing stool consistency [32,77,78]. Temporarily emphasizing soluble fiber (e.g., bananas, rice, applesauce, oats) may help improve stool form [130,131]. Limiting high-fat and greasy foods during symptomatic periods is advisable, as is reducing intake of sugar alcohol-containing products [48]. Although most gastrointestinal adverse effects are mild and self-limited, persistent or clinically significant symptoms may require dose adjustment or medical management, particularly when oral intake or hydration is compromised [16,56,90,137]. A summary of symptom-specific mechanisms and targeted nutrition strategies to improve tolerability is provided in Table 2.

**Table 2:** Symptom-targeted nutrition considerations that may support tolerability during incretin-based therapy.

Symptom	Likely Mechanism	Common Dietary Triggers	Targeted Nutrition Strategies	Escalate if Persistent or Severe
Nausea [16,27,56,72,73,124,125,126,127,128,135]	Delayed gastric emptying; gastric distension; dose escalation effects	Large meals; high-fat meals; rapid eating; dehydration; strong food odors	Small, structured meals; stop at early satiety; moderate fat per meal; slow eating; scheduled fluids between meals if tolerated; cold/room-temperature foods when helpful	Persistent symptoms limiting intake or hydration despite dietary modification
Vomiting/Reflux [16,27,56,72,73,101,124,125,126,136]	Reduced gastric clearance; increased gastric volume; LES relaxation	Large evening meals; high-fat foods; large fluid + meal combinations	Smaller evening meals; upright posture after eating; moderate fat; avoid over-distension	Recurrent vomiting, weight instability, or refractory reflux symptoms
Constipation [27,56,72,98,127,131,132]	Slowed transit; reduced intake volume; low hydration	Low fiber baseline; low fluid intake; sedentary behavior	Gradual fiber titration; emphasize soluble fiber when tolerated; optimize hydration; encourage movement; consider osmotic agents or medical management if persistent	No bowel movement >3–4 days, significant discomfort, or failure of dietary measures
Diarrhea [27,56,72,73,126,131,132,135,136]	Altered intestinal motility; fat sensitivity; bile acid changes	High-fat foods; spicy foods; sugar alcohols; excess caffeine	Emphasize soluble fiber; reduce dietary fat temporarily; limit sugar alcohols and irritants; gradual diet normalization	Persistent symptoms, dehydration, electrolyte disturbance, bleeding, fever, or inability to maintain intake
Bloating [27,56,72,125,131,132]	Fermentation of rapidly introduced fiber; slowed gastric emptying	Rapid fiber increase; large mixed meals; carbonated beverages	Gradual fiber increase; smaller meals; reduce carbonation; slow eating	Severe pain, progressive distension, vomiting, inability to pass stool/flatus, or concern for obstruction

Abbreviations: LES, lower esophageal sphincter. Note: Symptom-targeted strategies are based on incretin-specific adverse-event profiles, known effects on gastric emptying and intestinal motility, general gastrointestinal nutrition-management principles, and expert consensus. Direct randomized trials comparing specific dietary strategies for gastrointestinal symptom control during GLP-1 receptor agonist or dual incretin therapy remain limited.

## 6. Dietary Pattern and Macronutrient Approaches During Therapy

### 6.1. Protein Prioritization and Body Composition Monitoring

The substantial weight loss induced by GLP-1 receptor agonists and dual incretin therapies raises an important body-composition consideration: how to maximize fat-mass reduction while limiting excessive loss of fat-free mass [91]. Across pharmacologic weight-loss trials, reductions in fat-free mass typically account for approximately 20–40% of total weight lost, a proportion comparable to other hypocaloric interventions [91,138]. Available incretin-specific body-composition data suggest that weight loss with semaglutide and tirzepatide is driven predominantly by reductions in fat mass, although lean mass or fat-free mass also declines as part of total weight loss [16,139]. Given that excessive lean mass loss may reduce resting energy expenditure and impair physical function, clinical guidance commonly includes the active management of the rate of weight loss and the quality of intake. Obesity evidence-informed clinical considerations commonly recommend a moderate energy deficit (≈500–750 kcal/day) to support clinically meaningful weight loss while reducing the risk of disproportionate fat-free mass losses that are more likely with aggressive caloric restriction [140,141,142].

Appetite suppression and early satiety increase the risk of inadequate protein intake unless proactively addressed, particularly as dietary variety and total oral intake often decline during GLP-1RA dose escalation [44,143]. A recent clinical nutrition perspective highlights that early satiety and reduced dietary variety during GLP-1RA therapy often result in substantially lower oral intake, which can lead to inadequate nutrient intake and necessitates careful dietary planning [95]. However, randomized trials have not yet tested protein dose–response strategies specifically in patients receiving GLP-1RAs or dual GIP/GLP-1RAs. Accordingly, protein targets during incretin therapy should be interpreted as extrapolated from broader obesity, caloric restriction, aging, sarcopenia, and exercise-nutrition literature rather than as incretin-specific evidence-based thresholds [144,145].

Protein intake is therefore central to nutrition management during incretin therapy. Evidence from obesity and caloric restriction trials supports protein intakes in the range of approximately 1.2–1.6 g/kg/day (adjusted for age, renal function, and comorbidities) support fat-free mass retention and reduce the risk of inadequate intake during active weight reduction [146,147,148]. Individuals with sarcopenic obesity, low baseline muscle mass, or impaired strength may benefit from targeting the upper end of this range, combined with early initiation of resistance training, whereas metabolically healthy individuals with preserved muscle mass may remain closer to the lower end of this spectrum provided adequate mechanical stimulus is present [112,148,149,150]. These targets should be individualized according to age, renal function, baseline muscle mass, comorbidities, dietary tolerance, and treatment phase; in patients with chronic kidney disease or other conditions requiring protein restriction, higher-protein targets should not be applied without individualized clinical and dietetic assessment [112,148,149,150].

Importantly, muscle protein synthesis is optimized when protein intake is distributed across meals rather than consumed in a single large bolus [151]. Intakes of roughly 25–35 g of high-quality protein per eating occasion are generally sufficient to maximally stimulate muscle protein synthesis in most adults, particularly when leucine-rich sources are included [152]. This anabolic response is closely linked to a per-meal leucine threshold of approximately 2.5–3.0 g, typically achieved with ~25–35 g of high-quality protein from sources such as whey, dairy, eggs, lean poultry, fish, or soy. Suboptimal protein quality or highly uneven daily distribution may blunt muscle protein synthesis even when total daily protein intake appears adequate [153,154,155]. However, the per-meal leucine threshold has not been specifically validated as a clinical target during incretin-based obesity pharmacotherapy; therefore, leucine should be framed as a marker of high-quality protein intake rather than as a stand-alone supplementation requirement for all patients [153,154,155].

Because appetite is often markedly reduced, clinical practice models frequently emphasize prioritizing protein consumption at the beginning of meals to help ensure adequate intake before early satiety limits consumption [156]. This “protein-first” strategy is especially relevant in individuals consuming smaller meals or fewer daily eating occasions [156]. Nevertheless, it should be implemented flexibly and without reinforcing restrictive eating patterns, particularly in patients with a history of disordered eating or very low intake.

Practical meal-planning guidance may include anchoring each eating occasion around a high-quality protein source (e.g., Greek yogurt, cottage cheese, eggs, tofu/tempeh, fish, or poultry), followed by fiber-containing carbohydrates and cardioprotective fats in modest portions to maintain tolerability [157,158]. Randomized controlled trials of higher-protein diets during caloric restriction consistently demonstrate greater retention of fat-free mass compared with lower-protein diets, and meta-analyses indicate that protein supplementation combined with resistance training confers additive benefits for muscle mass and strength preservation, particularly in older adults and those at risk for sarcopenia [147,159]. These data support the biological plausibility and clinical rationale for protein prioritization during incretin therapy, but they remain largely indirect for GLP-1RA- or dual incretin-treated populations [144,160].

Meal timing may also contribute to anabolic signaling. Protein ingestion in the hours surrounding resistance training can enhance post-exercise muscle protein synthesis and lean mass adaptations, particularly in older adults, although the precise “anabolic window” is broad and total daily protein intake often exerts a stronger influence on long-term hypertrophy outcomes [161,162]. Caution is warranted with unsupervised time-restricted eating approaches, as shortened eating windows may inadvertently reduce total protein intake unless carefully structured [163]. Although this review focuses primarily on macronutrient strategies, clinicians should also consider adequate omega-3 fatty acid intake, and diets rich in antioxidant-containing fruits and vegetables may support overall musculoskeletal health within a Mediterranean-style framework [163,164,165]. Furthermore, emerging evidence suggests that, beyond total protein intake, protein quality, particularly leucine content, as well as adequate vitamin D status may contribute to the preservation of fat-free mass and muscle strength during weight loss [145]. Accordingly, supplementation strategies including branched-chain amino acids and vitamin D may be considered in selected patients, particularly in those at risk of sarcopenia or with documented deficiency. However, routine branched-chain amino acid or vitamin D supplementation should not be implied for all patients receiving incretin therapy; supplementation should be individualized according to dietary intake, deficiency status, sarcopenia risk, renal function, and clinical context.

Resistance training provides the complementary mechanical stimulus needed to support muscle strength, physical function, and fat-free mass retention during weight loss [164]. Randomized trials in hypocaloric states consistently demonstrate that resistance exercise performed two to three times per week attenuates lean mass loss, preserves fat-free mass, and improves functional outcomes during weight reduction. Trials combining caloric restriction with structured resistance training show greater preservation of lean mass and improvements in strength and mobility compared with diet-only interventions [66,165]. A meta-analysis of 49 randomized controlled trials involving prolonged resistance exercise training found that dietary protein supplementation significantly enhanced resistance training-induced increases in fat-free mass (mean difference 0.30 kg, 95% CI 0.09 to 0.52) and one-repetition-maximum strength (mean difference 2.49 kg, 95% CI 0.64 to 4.33) compared with resistance training alone, with diminishing additional benefit above ~1.6 g/kg/day total protein intake [145]. Another systematic review and meta-analysis in community-dwelling older adults with sarcopenia reported that combining protein supplementation with resistance exercise significantly increased muscle mass (standardized mean difference [SMD] 0.95, 95% CI 0.13–1.78) and muscle strength (SMD 0.32, 95% CI 0.08–0.56) compared with control conditions, supporting additive effects of nutritional and exercise interventions in this population [166].

These data strongly support the biological rationale for combining adequate protein intake with resistance training during weight reduction. However, most evidence comes from caloric restriction, aging, sarcopenia, and exercise-nutrition studies rather than randomized trials specifically conducted in GLP-1 receptor agonist or dual incretin-treated populations [66,145,165,167]. Accordingly, resistance training should be framed as a strongly evidence-informed extrapolation for patients receiving incretin therapy, rather than as an intervention already proven to improve body-composition outcomes in this specific pharmacologic context.

Pharmacotherapy can reduce appetite and energy intake, but it does not provide the mechanical loading stimulus required to maintain or improve muscle strength and functional capacity [166]. Aerobic exercise remains valuable for cardiometabolic health; however, resistance training appears central when the goal is supporting strength, physical function, and attenuation of excessive fat-free mass loss [114,168]. Concurrent training programs can be effective when appropriately balanced, avoiding excessive endurance volume that may compromise strength adaptations [169,170]. In the context of incretin therapy, the combination of sufficient protein intake and structured resistance training represents a clinically plausible and evidence-informed strategy to support healthier body composition and functional capacity during active weight reduction, while recognizing that direct incretin-specific exercise trials remain limited.

### 6.2. Dietary Pattern Selection and Macronutrient Quality

While incretin-based pharmacotherapy reduces total energy intake across a range of dietary patterns, dietary quality remains clinically relevant for cardiometabolic health, gastrointestinal tolerance, nutritional adequacy, and long-term sustainability [27,56,120]. Rather than prescribing rigid macronutrient ratios, evidence supports emphasizing dietary patterns that are nutrient-dense, digestively tolerable, and sustainable beyond the active pharmacotherapy phase [171,172].

A Mediterranean-style dietary pattern provides a strong evidence base for cardiometabolic benefit and long-term adherence [173,174,175,176,177]. Characterized by abundant vegetables, fruits, legumes, whole grains, fish, olive oil, and moderate dairy intake, this pattern aligns well with the reduced appetite and smaller meal volumes typical of incretin therapy [78,178,179]. A higher-protein Mediterranean variant may be particularly advantageous during active weight loss to support lean mass preservation while maintaining cardiovascular risk reduction [180,181].

Gut microbiota may represent an additional mechanistic link between dietary pattern quality, metabolic health, and inter-individual variability in response to incretin-based therapies. GLP-1RAs and changes in diet or weight loss may influence gut microbial composition, while microbial metabolites such as short-chain fatty acids and bile-acid derivatives may modulate endogenous incretin signaling [182]. However, most evidence remains mechanistic, preclinical, or observational, and causality in humans is not established. Therefore, microbiota-directed strategies should not currently be presented as specific clinical tools to enhance GLP-1 receptor agonist or dual incretin response. Instead, clinicians should emphasize dietary patterns already supported for cardiometabolic health and gastrointestinal function, including adequate fiber, legumes, fruits, vegetables, whole grains, and minimally processed foods, while recognizing microbiota modulation as an emerging research area [182,183].

Lower-carbohydrate approaches, including moderate carbohydrate restriction (<130 g/day), may improve glycemic control in individuals with type 2 diabetes and can be compatible with incretin therapy [184]. However, excessively restrictive carbohydrate intake may inadvertently reduce fiber consumption and increase the risk of constipation in the setting of delayed gastric emptying [98,99]. Careful attention to non-starchy vegetables, legumes, and other fiber-containing foods remains important.

Hybrid or adaptive dietary approaches, including ketogenic-Mediterranean patterns, have been proposed to counter reductions in energy expenditure during rapid weight loss. Controlled feeding and intervention studies suggest that nutritional ketosis or lower-carbohydrate diets may attenuate declines in resting metabolic rate or modestly increase energy expenditure during weight-loss phases, although findings remain heterogeneous and context dependent [185,186,187,188,189,190,191,192]. While short-term metabolic effects have been described in tightly controlled feeding studies, long-term comparative data in patients receiving incretin therapy remain limited. Accordingly, such approaches should be individualized and monitored to ensure adequate protein, fiber, and overall dietary quality.

Importantly, incretin therapy frequently reduces hedonic drive for ultra-processed and energy-dense foods, creating an opportunity to reshape long-standing dietary habits [193,194]. Evidence suggests that clinicians should leverage this period of reduced “food noise” to establish structured meal patterns and high-quality dietary practices that can be sustained if medication is tapered or discontinued [51,195]. Dietary strategies adopted during therapy should therefore prioritize sustainability and metabolic resilience, forming the foundation for long-term weight maintenance rather than serving as a temporary adjunct to pharmacologic appetite suppression. Long-term weight-loss research consistently demonstrates that durable outcomes are most strongly associated with sustained dietary adherence, high-quality eating patterns, and ongoing behavioral support rather than short-term macronutrient manipulation alone [195,196]. Studies further show that individuals who maintain weight loss over time are more likely to adhere to structured, nutrient-dense dietary patterns and consistent eating behaviors, underscoring the importance of establishing sustainable nutrition strategies during active treatment phases [197]. To support clinical translation, Table 3 summarizes key nutrition priorities during incretin-based therapy, integrating protein targets, fiber and hydration goals, meal structure, and dietary pattern recommendations.

## 7. Maintenance, Special Populations, and Implementation

### 7.1. Maintenance and Discontinuation Planning

Planning for weight maintenance and potential medication discontinuation is a critical yet often underdeveloped component of incretin-based obesity treatment [78]. Randomized withdrawal trials consistently demonstrate that cessation of GLP-1 or dual incretin therapy is followed by partial or substantial weight regain in many patients. In the STEP 1 extension, participants who discontinued semaglutide regained a mean of 11.6 percentage points of lost weight during the off-treatment extension, corresponding to approximately two-thirds of the prior weight loss. In SURMOUNT-4, withdrawal of tirzepatide after an initial treatment period led to substantial regain, whereas continued therapy maintained and augmented weight reduction [5,137]. In analyses from the STEP semaglutide program, participants regained a significant proportion of lost weight after treatment withdrawal compared with placebo, and in the SURMOUNT-4 tirzepatide withdrawal trial, those discontinuing therapy experienced marked recoupment of body weight that contrasted with continued weight maintenance on active treatment. Meta-analytic evidence further confirms that stopping GLP-1RA or dual agonist therapy results in rapid rebound of weight loss and attenuation of metabolic benefit [3,137,207]. These findings reinforce the chronic nature of obesity and highlight the need for structured long-term nutrition and behavioral strategies, regardless of whether pharmacotherapy is continued indefinitely or eventually tapered.

Although evidence remains limited regarding optimal discontinuation protocols, gradual dose reduction is commonly used in clinical practice to mitigate abrupt increases in appetite and caloric intake [208]. When gradual dose reduction is used in clinical practice, it may help patients adapt to returning hunger cues while reinforcing structured eating patterns and protein prioritization [27,77]. Some clinicians have also explored extended dosing intervals or lower “maintenance” doses once target weight is achieved; however, robust comparative trials evaluating these approaches are lacking [209]. A recent modeling analysis explored alternative dosing regimens of GLP-1RAs and suggested that reducing dosing frequency (e.g., from once weekly to once every two weeks) could maintain a large proportion (≈75%) of weight loss, but these findings should be considered hypothesis-generating rather than evidence of an established maintenance protocol [210]. Until stronger evidence emerges, discontinuation planning should emphasize reinforcement of sustainable dietary patterns, adequate protein intake, and continued physical activity.

Structured physical activity plays an important role in weight-loss maintenance following pharmacotherapy [64,68]. Exercise interventions after weight reduction have been shown to improve appetite regulation, support energy expenditure, and attenuate weight regain across obesity populations [68]. Emerging evidence also suggests that regular exercise may enhance endogenous GLP-1 responses and postprandial satiety signaling, providing a physiologic complement to pharmacologic therapy [211,212]. Establishing consistent resistance and aerobic exercise routines during active treatment may therefore help buffer against rebound hyperphagia if medication is reduced or discontinued [64].

### 7.2. Considerations for Special Populations

#### 7.2.1. Sex as a Biological Variable

Sex as a biological variable should be considered when implementing nutrition support during GLP-1 receptor agonist and dual incretin therapy. Although current evidence does not support sex-specific nutrition prescriptions during incretin-based obesity pharmacotherapy, several sex-related differences may influence treatment response, gastrointestinal tolerability, body-composition risk, and monitoring needs. Recent evidence suggests that women may experience slightly greater weight reduction than men during GLP-1 receptor agonist therapy, although the magnitude, mechanisms, and clinical implications of this difference remain incompletely defined [213,214].

Sex-related differences may be particularly relevant for nutrition care because appetite regulation, food reward, fat distribution, fat-free mass, and gastrointestinal symptom susceptibility can differ between men and women. Women generally have lower absolute fat-free mass than men, and sex differences in the composition of weight loss have been observed during dietary weight-loss interventions. These observations do not justify universal sex-specific protein targets during incretin therapy, but they support individualized assessment of baseline muscle mass, strength, protein intake, menopausal status when clinically relevant, and risk of low energy or nutrient intake during periods of marked appetite suppression [214,215].

Gastrointestinal tolerability may also warrant sex-aware monitoring. Some GLP-1 receptor agonist literature suggests sex differences in the frequency of adverse events, although findings are not fully consistent across trials and many studies are not powered for sex-specific safety analyses. Therefore, clinicians should avoid assuming uniform tolerability across patients and should monitor nausea, vomiting, constipation, hydration, and intake adequacy in a patient-centered manner during dose escalation [214].

At present, there are no randomized trials testing sex-specific nutrition strategies during GLP-1 receptor agonist or dual incretin therapy. Accordingly, the practical implications of sex as a biological variable are primarily related to risk identification, individualized monitoring, and research design rather than to different prescriptive dietary algorithms. Future studies should report sex-stratified outcomes for dietary intake, protein adequacy, gastrointestinal symptom burden, dose-escalation success, treatment persistence, fat-free mass, muscle strength, physical function, discontinuation, and post-discontinuation weight regain.

#### 7.2.2. Chronic Kidney Disease

GLP-1RAs are generally safe for individuals with chronic kidney disease (CKD) and have demonstrated renoprotective effects in large cardiovascular and renal outcomes trials, including reductions in albuminuria and slower decline in estimated glomerular filtration rate [216]. A recent comprehensive review concluded that GLP-1RAs may slow CKD progression by improving glycemic control, reducing albuminuria, and possibly protecting against glomerular damage, with evidence from trials such as SUSTAIN-6, REWIND, and the dedicated FLOW CKD trial [217]. Meta-analyses focusing on CKD populations report that GLP-1 RA treatment is associated with a reduced risk of substantial eGFR decline and composite kidney outcomes in people with impaired renal function [218]. Because these agents are not primarily cleared by the kidneys, dose adjustment is often unnecessary in mild-to-moderate CKD [219]. However, GI side effects such as nausea, vomiting, or reduced fluid intake may predispose vulnerable patients to dehydration and acute kidney injury [220,221]. Careful attention to hydration and monitoring of renal function during dose escalation is therefore warranted. Protein recommendations in CKD require individualized adjustment based on disease stage and clinical goals [221]. While higher protein intake supports lean mass preservation during weight loss, excessive intake may be inappropriate in advanced CKD [206]. Collaboration with a renal dietitian can help balance protein adequacy with renal protection in this population [206].

#### 7.2.3. Metabolic Dysfunction-Associated Steatotic Liver Disease

Weight reduction remains the cornerstone of therapy for metabolic dysfunction-associated steatotic liver disease (MASLD) and steatohepatitis [75,222]. Clinical trials of GLP-1RAs and dual incretin therapies demonstrate significant reductions in hepatic steatosis and increasing rates of steatohepatitis resolution with pharmacologically induced weight loss. In a phase 2 randomized trial, semaglutide achieved histologic resolution of steatohepatitis without fibrosis worsening in a substantially greater proportion of patients than placebo, and subsequent trials and meta-analyses have confirmed improvements in liver fat content, inflammation, and metabolic dysfunction-associated steatotic liver disease outcomes [223,224,225]. More recent randomized studies of dual GIP/GLP-1 agonists similarly report marked reductions in liver fat and higher rates of steatohepatitis resolution compared with placebo [223]. Evidence suggests that weight loss of approximately 7–10% is associated with improvement in steatohepatitis, while reductions exceeding 10% may be required for fibrosis improvement [226]. Nutrition strategies should therefore prioritize sustainable caloric reduction, dietary quality, and cardiometabolic risk reduction, with emphasis on Mediterranean-style or similarly cardioprotective patterns that support hepatic and metabolic health [75,200,227,228].

### 7.3. Implementation and Clinic Workflow

The rapid expansion of incretin-based pharmacotherapy has created new demands on clinical workflows, requiring structured, multidisciplinary approaches to ensure safe and effective long-term management. Programs that integrate obesity medicine clinicians, registered dietitians, and behavioral health professionals are best positioned to address the physiological, nutritional, and behavioral determinants of treatment success [95,229]. Nutrition support is particularly critical during early dose escalation and during transitions to maintenance or discontinuation phases [27,77,90].

Frequent follow-up during the initiation phase improves tolerability and adherence. In the absence of comparative trials defining optimal follow-up intensity during incretin-based obesity pharmacotherapy, visit frequency should be individualized according to dose-escalation phase, symptom burden, nutritional risk, comorbidities, and access to care [208,209]. Once a stable maintenance dose is achieved, follow-up intervals can typically be extended to every three to six months, with interim contact as needed for symptom management or weight changes [230]. Telehealth delivery models, asynchronous nutrition support, and remote monitoring tools may improve access and continuity of care, but their effectiveness specifically among patients receiving GLP-1 receptor agonist or dual incretin therapy requires further evaluation. These approaches should therefore be viewed as implementation strategies with emerging relevance rather than established consensus standards [27,231,232,233].

Remote patient monitoring tools may further enhance care delivery. Connected scales and digital monitoring platforms allow clinicians to track weight trajectories in real time, improving engagement and retention while enabling early identification of clinically meaningful changes that may signal excessive caloric restriction, dehydration, or emerging nonadherence. Studies of digital weight-management programs show that provision of connected scales and remote monitoring increases frequency of self-weighing, improves retention, and facilitates timely clinical feedback, all of which are associated with greater likelihood of achieving clinically significant weight loss and sustaining behavioral adherence [234]. Early identification of unintended rapid weight loss or regain enables timely dietary counseling, adjustment of medication dosing, or reinforcement of behavioral strategies [235,236]. It is important to recognize that not all patients have access to or are comfortable with digital technologies (barriers including limited digital literacy, connectivity, or device availability can impede engagement) and low-tech alternatives such as paper food logs, telephone check-ins, and manual self-monitoring remain valuable strategies for supporting adherence and dietary behavior change in these populations [237,238].

**Figure 3:**
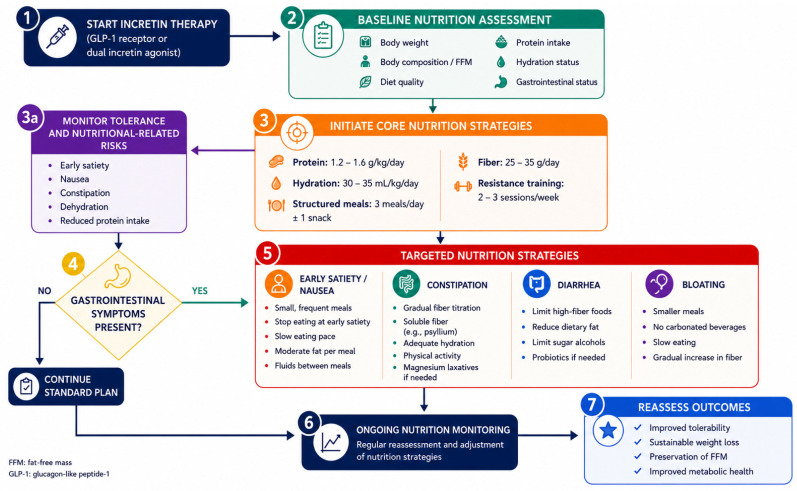
Proposed Clinical Decision Framework for Nutrition Management During Incretin Therapy. This clinical algorithm illustrates a nutrition-centered approach to support patients treated with GLP-1RAs or dual incretin therapies for obesity. The framework begins with a baseline nutrition assessment evaluating body weight, body composition, diet quality, protein intake, hydration status, and gastrointestinal history. Core nutrition strategies, including adequate protein intake, hydration, gradual fiber intake, structured meals, and resistance training, may be considered to support nutritional adequacy, body-composition monitoring, and clinical decision-making during therapy. Ongoing monitoring of gastrointestinal tolerability may support early identification of common symptoms such as early satiety, nausea, constipation, diarrhea, and bloating. When symptoms occur, targeted nutrition strategies are applied to improve tolerability while maintaining adequate nutrient intake. Continuous monitoring and reassessment are intended to support treatment persistence, nutritional adequacy, symptom management, body-composition monitoring, and long-term weight-maintenance planning. Abbreviations: GLP-1, glucagon-like peptide-1; FFM, fat-free mass.

Emerging practice models commonly use more frequent contact during early dose escalation to address gastrointestinal symptoms, reinforce protein and hydration targets, and identify inadequate intake or rapid unintended weight loss. Once patients are clinically stable, follow-up intervals may be extended, although the optimal schedule remains uncertain. The practical application of these principles is summarized in the clinical decision algorithm presented in Figure 3.

## 8. Limitations and Evidence Gaps

This review should be interpreted in light of several limitations. Although a structured literature search was conducted, the article was designed as a narrative review rather than a systematic review or meta-analysis. Therefore, no formal risk-of-bias assessment, certainty grading, or quantitative evidence synthesis was performed. The purpose of the review was to integrate available pharmacological, nutritional, gastrointestinal, body-composition, and implementation evidence into a clinically operational framework, rather than to generate formal graded recommendations.

Direct evidence evaluating structured nutrition interventions specifically in patients receiving GLP-1RAs or dual GIP/GLP-1RAs for obesity remains limited [27,30,44]. Most randomized incretin trials provide robust data on weight loss, cardiometabolic outcomes, gastrointestinal adverse events, and treatment withdrawal, but they generally do not test specific nutrition strategies such as protein dose targets, meal timing, fiber titration, hydration protocols, or symptom-directed dietary algorithms. Accordingly, several recommendations in this review are extrapolated from the broader obesity, caloric restriction, body-composition, sarcopenia, resistance-training, bariatric, and gastrointestinal symptom-management literature. These recommendations are biologically plausible and clinically relevant, but they should be interpreted as evidence-informed clinical considerations rather than as interventions proven to improve medication persistence, reduce adverse-event rates, or prevent weight regain in incretin-treated populations.

The optimal protein intake, protein distribution, leucine threshold, meal frequency, dietary pattern, and macronutrient composition during incretin-based therapy remain uncertain. Protein targets such as 1.2–1.6 g/kg/day and per-meal high-quality protein goals are supported primarily by indirect evidence from the weight-loss, exercise nutrition, aging, and sarcopenia literature [144,147,160]. Whether these targets improve lean-mass retention, physical function, treatment adherence, or long-term weight maintenance specifically during GLP-1 receptor agonist or dual incretin therapy has not been adequately tested in randomized dose–response trials. Similarly, although resistance training is strongly supported by the broader weight-loss and aging literature, its additive effect during incretin-induced weight loss requires further prospective evaluation using body-composition and functional endpoints.

Several implementation strategies discussed in this review, including early nutrition follow-up, symptom monitoring, telehealth support, remote weight tracking, tapering approaches, and extended dosing intervals, should be considered emerging practice models rather than established consensus evidence-informed clinical considerations. These strategies may be useful in clinical practice, particularly for patients with gastrointestinal symptoms, low intake, rapid weight loss, or high risk of discontinuation, but comparative trials are needed to determine their effectiveness, feasibility, cost-effectiveness, and equity across different health systems.

Important population-specific evidence gaps remain. Sex as a biological variable is particularly underexplored in nutrition support during incretin therapy, despite known sex-related differences in appetite regulation, body composition, gastrointestinal symptom prevalence, and vulnerability to lean-mass loss during weight reduction [213,214,215]. Future studies should report sex-stratified outcomes for gastrointestinal tolerability, dietary intake, body composition, discontinuation, and weight regain. Additional research is also needed in older adults, individuals with sarcopenic obesity, patients with prior metabolic or bariatric surgery, chronic kidney disease, metabolic dysfunction-associated steatotic liver disease, and individuals with restrictive or binge-eating risk. In these groups, nutrition recommendations may require closer individualization, but direct evidence remains insufficient to support population-specific prescriptions beyond careful monitoring and multidisciplinary care.

Future research should move beyond descriptive frameworks and test structured nutrition-supported incretin care models in pragmatic clinical trials. Priority endpoints should include gastrointestinal symptom burden, medication persistence, dose-escalation success, dietary adequacy, protein intake, micronutrient status, hydration, fat-free mass, muscle strength, physical function, quality of life, treatment discontinuation, and post-discontinuation weight regain. Such studies should distinguish direct pharmacological effects from the added value of nutrition care and should evaluate whether tailored nutrition support improves clinically meaningful outcomes beyond standard pharmacotherapy follow-up.

### Clinical Take-Home Messages

The following take-home messages summarize pragmatic clinical considerations derived from direct incretin evidence where available and from the indirect nutrition, gastrointestinal, body-composition, and expert-consensus literature where direct evidence is lacking. First, nutrition care should begin before or at the initiation of incretin-based therapy, because appetite suppression, early satiety, and gastrointestinal symptoms can reduce meal size, dietary variety, protein intake, fluid intake, and fiber tolerance. Second, protein, hydration, fiber, and meal structure should be individualized during dose escalation, with particular attention to patients with low baseline intake, gastrointestinal symptoms, older age, sarcopenic obesity, chronic kidney disease, prior metabolic or bariatric surgery, or restrictive/disordered eating risk. Third, gastrointestinal symptoms are often meal-related and may improve with smaller portions, slower eating pace, moderate dietary fat, scheduled hydration, and gradual fiber titration; however, persistent or severe symptoms require medical reassessment. Fourth, protein and resistance-training strategies are clinically relevant for body-composition monitoring, but their targets are largely extrapolated from the broader weight-loss, aging, sarcopenia, and exercise-nutrition literature rather than incretin-specific nutrition trials. Fifth, maintenance planning should begin during active weight loss, particularly for patients at risk of treatment interruption, because medication discontinuation is commonly followed by partial or substantial weight regain.

## 9. Conclusions

The emergence of GLP-1RAs and dual incretin therapies has transformed the therapeutic landscape of obesity management, enabling levels of weight reduction previously achievable primarily through metabolic and bariatric surgery. However, the long-term effectiveness of these agents in real-world clinical practice depends not only on pharmacologic potency but also on the integration of structured nutrition and lifestyle support. GI tolerability, preservation of lean body mass, and sustainability of weight loss are all strongly influenced by dietary patterns, protein intake, hydration, and behavioral adaptation during therapy.

A nutrition-first framework provides a practical strategy to align dietary guidance with the physiological effects of incretin-based therapies. Symptom-responsive meal structuring, protein prioritization, hydration and fiber management, and resistance-training support may help address common clinical challenges during treatment, including reduced intake, gastrointestinal symptom burden, and body-composition risk. However, direct evidence that structured nutrition interventions improve medication persistence, reduce adverse-event rates, or prevent weight regain in incretin-treated populations remains limited. The contribution of this review is therefore primarily conceptual and translational. It organizes heterogeneous evidence intro practical domains for assessment, counseling, monitoring, and future research rather than establishing validated nutrition protocols for incretin-treated populations. These strategies should therefore be interpreted as evidence-informed components of multidisciplinary obesity care and as priorities for future pragmatic trials. Equally important, establishing sustainable dietary patterns and resistance training habits during active pharmacotherapy creates a foundation for long-term weight maintenance and metabolic health, particularly if treatment is interrupted or discontinued.

The pre-treatment assessment framework (Table 1) and symptom-targeted strategies (Table 2) provide practical, translatable clinical tools that can be evaluated in pragmatic trials. As the use of incretin-based therapies continues to expand, future research should prioritize these trials and implementation studies to test nutrition-supported care models, identify maintenance strategies, and assess outcomes across diverse patient populations. Embedding structured nutrition care within multidisciplinary obesity treatment pathways represents a promising approach to supporting the translation of pharmacologic advances into durable, clinically meaningful benefits for individuals living with obesity, but its impact should be evaluated in pragmatic trials and implementation studies.

## Figures and Tables

**Table 1:** Pre-treatment nutrition assessment to optimize tolerability and adherence to incretin-based therapy.

	What to Assess	Clinical Relevance During Therapy	Practical Action
Overall Diet Quality [27,44,68,83]	Ultra-processed food reliance; protein density; high-fat meal frequency; fruit/vegetable intake	Low-quality, high-fat patterns increase GI intolerance and dietary inadequacy once intake declines	Shift toward protein-dense, nutrient-rich pattern; reduce large/high-fat meals; establish structured eating
Protein Intake and Distribution [27,49,68,70,78,91,92]	Total grams/day; meal distribution; breakfast protein; tolerance to solid protein	Appetite suppression may reduce protein intake below levels commonly recommended during active weight loss; targets are extrapolated from broader weight-loss and body-composition literature	Set minimum protein target; distribute across meals; prioritize high-quality protein sources
Baseline Muscle Mass and Function [112,113,114,115,116,117]	Bioimpedance or DXA (if available); handgrip strength; chair-stand performance	Identifies sarcopenic obesity and patients who may be at higher risk of functional decline or excessive fat-free mass loss during rapid weight reduction	Prioritize higher protein targets; initiate resistance training early; monitor functional performance
Hydration Status [56,70,78,118,119]	Total fluid intake; prolonged gaps without fluids; caffeine-heavy intake	Low fluid intake may worsen nausea, fatigue, constipation, and dehydration risk during periods of reduced intake	Implement scheduled hydration; adjust timing if reflux occurs
Fiber Intake and Bowel Pattern [27,56,78,90,98]	Baseline fiber intake; stool frequency; constipation or IBS history	Slowed gastric emptying increases constipation risk	Gradual fiber titration; optimize hydration; monitor bowel changes
Meal Timing and Portion Pattern [27,30,90]	Skipped meals; large evening meals; rapid eating	Large, infrequent meals poorly tolerated with delayed gastric emptying	Encourage smaller structured meals; slow eating pace; avoid large late meals
Pre-existing GI Symptoms [27,70,90]	Reflux, chronic constipation, bloating, fat intolerance	Baseline GI dysfunction predicts early intolerance and dose reduction	Preemptive symptom strategies; moderate fat per meal; individualized titration pacing
Restrictive or Disordered Eating Risk [106,107,108,111]	History of binge eating or restrictive dieting; weight suppression; screening tools	History of binge eating, restrictive dieting, weight suppression, compensatory behaviors, or prior eating disorder treatment; screening tools	Use screening for risk identification and care planning; establish minimum intake targets; monitor closely; involve multidisciplinary support when risk is identified

Abbreviations: DXA, dual-energy X-ray absorptiometry; GI, gastrointestinal; IBS, irritable bowel syndrome. Note: The assessment domains in this table are intended to identify modifiable nutrition-related risks before and during incretin therapy. Their rationale integrates direct evidence on appetite suppression, reduced intake, and gastrointestinal adverse events during incretin treatment with indirect evidence from obesity nutrition care, body-composition, gastrointestinal, and expert-consensus literature. These actions should be interpreted as evidence-informed clinical considerations rather than formally graded recommendations.

**Table 3:** Evidence-Informed Nutrition Priorities to Consider During Incretin-Based Therapy.

	Target	Clinical Purpose	Practical Focus
Protein (daily) [27,146,147,148]	1.2–1.6 g/kg/day *	Support protein adequacy and help attenuate excessive fat-free mass loss during active weight reduction	Set minimum target; individualize for age/CKD/sarcopenia risk
Protein (per meal) [27,153,154,155]	25–35 g high-quality protein	Support muscle protein synthesis using high-quality protein sources; evidence extrapolated from exercise, aging, and sarcopenia literature	Anchor meals around dairy, eggs, fish, poultry, soy
Fiber [27,130,132,170,197,198,199]	~25–35 g/day (titrate gradually)	Reduce constipation risk with slowed gastric emptying	Emphasize soluble fiber; adjust to tolerance
Hydration [27,56,127,131]	~1.5–2.5 L/day (individualized)	Prevent nausea, fatigue, constipation	Scheduled sipping; separate large fluids from meals if needed
Meal Structure [27,72,163]	3 structured meals ± 1 protein snack	Support intake adequacy and meal-related GI tolerability; reduce risk of under-eating	Small portions; protein-first; moderate fat per meal
Dietary Pattern [85,89,173,175,178,180,181,200,201]	Mediterranean or higher-protein Mediterranean	Cardiometabolic benefit + sustainability	Vegetables, legumes, fish, olive oil; limit ultra-processed foods
Exercise Synergy [93,114,162,164,165,202,203]	Resistance training 2–3 times//week	Support strength, physical function, and attenuation of excessive fat-free mass loss; evidence primarily extrapolated from broader weight-loss and resistance-training literature	Combine with adequate protein; moderate endurance volume

* Protein targets should be individualized according to renal function, age, and overall clinical status, consistent with established clinical nutrition recommendations [204,205,206]. Abbreviations: CKD, chronic kidney disease. Note: Protein and resistance-training targets are extrapolated primarily from caloric restriction, exercise nutrition, aging, sarcopenia, and body-composition literature. Randomized trials testing protein dose–response or resistance-training prescriptions specifically during GLP-1 receptor agonist or dual incretin therapy remain limited. Targets should therefore be individualized according to age, renal function, baseline muscle mass, functional status, gastrointestinal tolerability, comorbidities, and treatment phase.

## Data Availability

No new data were created or analyzed in this study. Data sharing is not applicable to this article.

## References

[1] Frías J.P. (2020). Tirzepatide: A glucose-dependent insulinotropic polypeptide (GIP) and glucagon-like peptide-1 (GLP-1) dual agonist in development for the treatment of type 2 diabetes. Expert Rev. Endocrinol. Metab..

[2] DE Oca A.P.-M., Pellitero S., Puig-Domingo M. (2021). Obesity and GLP-1. Minerva Endocrinol..

[3] Quarenghi M., Capelli S., Galligani G., Giana A., Preatoni G., Quarenghi R.T. (2025). Weight Regain After Liraglutide, Semaglutide or Tirzepatide Interruption: A Narrative Review of Randomized Studies. J. Clin. Med..

[4] Wen J., How-Volkman C., Truong A., Nadora D., Bernstein E.M., Akhtar M., Puglisi J., Frezza E. (2024). Comparative Efficacy of Semaglutide Versus Liraglutide or Efinopegdutide on Weight Loss in Obese Patients: A Systematic Review and Meta-Analysis. Cureus.

[5] Aronne L.J., Horn D.B., le Roux C.W., Ho W., Falcon B.L., Valderas E.G., Das S., Lee C.J., Glass L.C., Senyucel C. (2025). Tirzepatide as Compared with Semaglutide for the Treatment of Obesity. N. Engl. J. Med..

[6] Jensen T.L., Brønden A., Karstoft K., Sonne D.P., Christensen M.B. (2024). The Body weight Reducing Effects of Tirzepatide in People with and without Type 2 Diabetes: A Review on Efficacy and Adverse Effects. Patient Prefer. Adherence.

[7] Jastreboff A.M., Aronne L.J., Ahmad N.N., Wharton S., Connery L., Alves B., Kiyosue A., Zhang S., Liu B., Bunck M.C. (2022). Tirzepatide Once Weekly for the Treatment of Obesity. N. Engl. J. Med..

[8] Bergmann N.C., Davies M.J., Lingvay I., Knop F.K. (2023). Semaglutide for the treatment of overweight and obesity: A review. Diabetes Obes. Metab..

[9] McGowan B., Ciudin A., Baker J.L., Busetto L., Dicker D., Frühbeck G., Goossens G.H., Monami M., Sbraccia P., Martinez-Tellez B. (2025). A systematic review and meta-analysis of the efficacy and safety of pharmacological treatments for obesity in adults. Nat. Med..

[10] Webb V.L., Wadden T.A. (2017). Intensive Lifestyle Intervention for Obesity: Principles, Practices, and Results. Gastroenterology.

[11] Wadden T.A., Chao A.M., Machineni S., Kushner R., Ard J., Srivastava G., Halpern B., Zhang S., Chen J., Bunck M.C. (2023). Tirzepatide after intensive lifestyle intervention in adults with overweight or obesity: The SURMOUNT-3 phase 3 trial. Nat. Med..

[12] Muralidharan J., Romain C., Martínez-Noguera F.J., Marín-Cascales E., Chung L., Alcaraz P., Cases J. (2026). A 16-week supplementation with a polyphenol-rich supplement, Sinetrol ^®^ Xpur, aids in fat loss of overweight and obese volunteers: A randomised, double-blind, parallel trial. Int. J. Food Sci. Nutr..

[13] Morán L.J., Aparicio V.A., Flor-Alemany M., Fernández-Bergés D., Nestares T., Nebot-Valenzuela E., Felix-Redondo F.J. (2025). The influence of Mediterranean diet and physical activity-related energy expenditure on weight status and cardiometabolic risk. What “weights” more? The HERMEX study. Int. J. Food Sci. Nutr..

[14] Biter L.U., ‘t Hart J.W., Noordman B.J., Smulders J.F., Nienhuijs S., Dunkelgrün M., Zengerink J.F., Birnie E., Friskes I.A., Mannaerts G.H. (2024). Long-term effect of sleeve gastrectomy vs Roux-en-Y gastric bypass in people living with severe obesity: A phase III multicentre randomised controlled trial (SleeveBypass). Lancet Reg. Health-Eur..

[15] O’brien P.E., Hindle A., Brennan L., Skinner S., Burton P., Smith A., Crosthwaite G., Brown W. (2019). Long-Term Outcomes After Bariatric Surgery: A Systematic Review and Meta-analysis of Weight Loss at 10 or More Years for All Bariatric Procedures and a Single-Centre Review of 20-Year Outcomes After Adjustable Gastric Banding. Obes. Surg..

[16] Wilding J.P.H., Batterham R.L., Calanna S., Davies M., Van Gaal L.F., Lingvay I., McGowan B.M., Rosenstock J., Tran M.T., Wadden T.A. (2021). Once-Weekly Semaglutide in Adults with Overweight or Obesity. N. Engl. J. Med..

[17] Reytor-González C., Campuzano-Donoso M., Sarno G., Montalvan M., Horowitz R., Rossetti G., Pilone V., Barrea L., Muscogiuri G., Schiavo L. (2026). Single vs. Dual Agonist Pharmacotherapy for Managing Insufficient Weight Loss and Weight Regain Following Metabolic and Bariatric Surgery: A Comparative Review. Nutrients.

[18] Aslam B., BIN Zafar M.D., Changez M.I.K., Abdullah M., Safwan M., Qamar B., Shinwari A., Rai S. (2025). Exploring the potential impact of GLP-1 receptor agonists in cancer therapy. Minerva Endocrinol..

[19] Yılmaz N., Bastemir M. (2026). Gastrointestinal Adverse Effects of GLP-1 and Dual GLP-1/GIP Receptor Agonists: A Comprehensive Update in Diabetic and Obese Populations. Diabetes Metab. Syndr. Obes. Targets Ther..

[20] Rodriguez P.J., Zhang V., Gratzl S., Do D., Cartwright B.G., Baker C., Gluckman T.J., Stucky N., Emanuel E.J. (2025). Discontinuation and Reinitiation of Dual-Labeled GLP-1 Receptor Agonists Among US Adults with Overweight or Obesity. JAMA Netw. Open.

[21] Sarno G., Reytor-González C., Frias-Toral E., Campuzano-Donoso M., Katsanos C.S., Simancas-Racines D. (2025). Navigating the Weight: The Impact of Obesity on Gastrointestinal Cancer Surgery and Strategies for Improved Outcomes. Semin. Cancer Biol..

[22] Simancas-Racines D., Reytor-González C., Frias-Toral E., Katsanos C.S., Hidalgo R. (2025). Weighty matters: Unraveling the impact of obesity on colorectal cancer and nutritional interventions. Semin. Cancer Biol..

[23] Kantilafti M., Magiakou E., Chrysostomou S. (2025). Ultra-processed foods and cancer risk: A narrative review of epidemiological findings and biological mechanisms. Int. J. Food Sci. Nutr..

[24] De Silva A., Bloom S.R. (2012). Gut Hormones and Appetite Control: A Focus on PYY and GLP-1 as Therapeutic Targets in Obesity. Gut Liver.

[25] Pratama K.G., Nugroho H., Hengky A., Tandry M., Pauliana P. (2024). Glucagon-like peptide-1 receptor agonists for post-bariatric surgery weight regain and insufficient weight loss: A systematic review. Obes. Med..

[26] Kolli R.T., Aoutla S., Jyothi N., Kalifa M.R.H.M., Raju A., Muralidharan K.C. (2025). Rebound or Retention: A Meta-Analysis of Weight Regain After the Discontinuation of Glucagon-Like Peptide-1 (GLP-1) Receptor Agonists and Other Anti-obesity Drugs. Cureus.

[27] Mozaffarian D., Agarwal M., Aggarwal M., Alexander L., Apovian C.M., Bindlish S., Bonnet J., Butsch W.S., Christensen S., Gianos E. (2025). Nutritional priorities to support GLP-1 therapy for obesity: A joint Advisory from the American College of Lifestyle Medicine, the American Society for Nutrition, the Obesity Medicine Association, and The Obesity Society. Am. J. Clin. Nutr..

[28] Sibal R., Balamurugan G., Langley J., Graham Y., Mahawar K. (2025). Macronutrient, Micronutrient Supplementation and Monitoring for Patients on GLP-1 Agonists: Can We Learn from Metabolic and Bariatric Surgery?. Nutrients.

[29] Shankar A., Sharma A., Vinas A., Chilton R.J. (2024). GLP-1 receptor agonists and delayed gastric emptying: Implications for invasive cardiac interventions and surgery. Cardiovasc. Endocrinol. Metab..

[30] Sievenpiper J., Ard J., Blüher M., Chen W., Dixon J., Fitch A., Gigliotti L., Khunti K., Lecube A., Lean M. (2026). Nutritional and lifestyle supportive care recommendations for management of obesity with GLP-1—Based therapies: An expert consensus statement using a modified Delphi approach. Obes. Pillars.

[31] García-Gorrita C., Onofre N.S., Merino-Torres J.F., Soriano J.M. (2025). Beyond GLP-1 Agonists: An Adaptive Ketogenic–Mediterranean Protocol to Counter Metabolic Adaptation in Obesity Management. Nutrients.

[32] van Gassel R.J., Bels J.L., van de Poll M.C. (2023). Nutritional strategies during gastrointestinal dysfunction. Curr. Opin. Crit. Care.

[33] Reiss A.B., Gulkarov S., Lau R., Klek S.P., Srivastava A., Renna H.A., De Leon J. (2025). Weight Reduction with GLP-1 Agonists and Paths for Discontinuation While Maintaining Weight Loss. Biomolecules.

[34] Choi W., Woo G.H., Kwon T.-H., Jeon J.-H. (2025). Obesity-Driven Metabolic Disorders: The Interplay of Inflammation and Mitochondrial Dysfunction. Int. J. Mol. Sci..

[35] Seino Y., Fukushima M., Yabe D. (2010). GIP and GLP-1, the two incretin hormones: Similarities and differences. J. Diabetes Investig..

[36] Tongta S., Sungkaworn T., Pathomthongtaweechai N. (2025). Neurobiological Mechanisms and Therapeutic Potential of Glucagon-like Peptide-1 Receptor Agonists in Binge Eating Disorder: A Narrative Review. Int. J. Mol. Sci..

[37] Alharbi A.G. (2025). GLP-1 receptor agonism: A transformative approach for managing type-2 diabetes and obesity. Saudi Pharm. J..

[38] Liu Q.K. (2024). Mechanisms of action and therapeutic applications of GLP-1 and dual GIP/GLP-1 receptor agonists. Front. Endocrinol..

[39] Yao H., Zhang A., Li D., Wu Y., Wang C.-Z., Wan J.-Y., Yuan C.-S. (2024). Comparative effectiveness of GLP-1 receptor agonists on glycaemic control, body weight, and lipid profile for type 2 diabetes: Systematic review and network meta-analysis. BMJ.

[40] Jalleh R.J., Plummer M.P., Marathe C.S., Umapathysivam M.M., Quast D.R., Rayner C.K., Jones K.L., Wu T., Horowitz M., Nauck M.A. (2024). Clinical Consequences of Delayed Gastric Emptying With GLP-1 Receptor Agonists and Tirzepatide. J. Clin. Endocrinol. Metab..

[41] Skibicka K.P. (2013). The central GLP-1: Implications for food and drug reward. Front. Neurosci..

[42] Park J.S., Kim K.S., Choi H.J. (2025). Glucagon-Like Peptide-1 and Hypothalamic Regulation of Satiation: Cognitive and Neural Insights from Human and Animal Studies. Diabetes Metab. J..

[43] Dang V., Sambuco N., Yammine L., Versace F. (2026). Do GLP-1 Receptor Agonists Alter Brain Responses to Reward-Related Cues? A Systematic Review. bioRxiv.

[44] Christensen S., Robinson K., Thomas S., Williams D.R. (2024). Dietary intake by patients taking GLP-1 and dual GIP/GLP-1 receptor agonists: A narrative review and discussion of research needs. Obes. Pillars.

[45] Hayashi D., Edwards C., Emond J.A., Gilbert-Diamond D., Butt M., Rigby A., Masterson T.D. (2023). What Is Food Noise? A Conceptual Model of Food Cue Reactivity. Nutrients.

[46] Johnson B., Milstead M., Thomas O., McGlasson T., Green L., Kreider R., Jones R. (2025). Investigating nutrient intake during use of glucagon-like peptide-1 receptor agonist: A cross-sectional study. Front. Nutr..

[47] Steger F.L., Jamshed H., Martin C.K., Richman J.S., Bryan D.R., Hanick C.J., Salvy S., Warriner A.H., Peterson C.M. (2022). Impact of early time-restricted eating on diet quality, meal frequency, appetite, and eating behaviors: A randomized trial. Obesity.

[48] Bina J.D., Tonsor G.T., Richards T.J. (2026). GLP-1 use and protein demand. Food Policy.

[49] Johnson B.V., Milstead M., Green L., Kreider R., Jones R. (2025). Diet quality and nutrient distribution while using glucagon-like-peptide-1 receptor agonist: A secondary cross-sectional analysis. Obes. Pillars.

[50] Nguyen T.L., Trinh K.S. (2021). Evaluation of the Obesity Prevention, Blood Glucose, and Blood Lipid Control of Vietnamese Rice Varieties in High-Fat Diet-Induced Obese Mice. Int. J. Food Sci..

[51] Cheney C., Hunter K., Klein M. (2025). Impact of GLP-1 Receptor Agonists on Perceived Eating Behaviors in Response to Stimuli. Diabetes Metab. Syndr. Obes. Targets Ther..

[52] Kim J.A., Yoo H.J. (2025). Exploring the Side Effects of GLP-1 Receptor Agonist: To Ensure Its Optimal Positioning. Diabetes Metab. J..

[53] Al Mushref M., Srinivasan S. (2013). Effect of high fat-diet and obesity on gastrointestinal motility. Ann. Transl. Med..

[54] Shu Y., He X., Wu P., Liu Y., Ding Y., Zhang Q. (2022). Gastrointestinal adverse events associated with semaglutide: A pharmacovigilance study based on FDA adverse event reporting system. Front. Public Health.

[55] Gentinetta S., Sottotetti F., Manuelli M., Cena H. (2024). Dietary Recommendations for the Management of Gastrointestinal Symptoms in Patients Treated with GLP-1 Receptor Agonist. Diabetes Metab. Syndr. Obes. Targets Ther..

[56] Gorgojo-Martínez J.J., Mezquita-Raya P., Carretero-Gómez J., Castro A., Cebrián-Cuenca A., de Torres-Sánchez A., García-De-Lucas M.D., Núñez J., Obaya J.C., Soler M.J. (2022). Clinical Recommendations to Manage Gastrointestinal Adverse Events in Patients Treated with Glp-1 Receptor Agonists: A Multidisciplinary Expert Consensus. J. Clin. Med..

[57] Valicente V.M., Peng C.-H., Pacheco K.N., Lin L., Kielb E.I., Dawoodani E., Abdollahi A., Mattes R.D. (2023). Ultraprocessed Foods and Obesity Risk: A Critical Review of Reported Mechanisms. Adv. Nutr. Int. Rev. J..

[58] Samms R.J., Coghlan M.P., Sloop K.W. (2020). How May GIP Enhance the Therapeutic Efficacy of GLP-1?. Trends Endocrinol. Metab..

[59] Mohammad S., Ramos L.S., Buck J., Levin L.R., Rubino F., McGraw T.E. (2011). Gastric Inhibitory Peptide Controls Adipose Insulin Sensitivity via Activation of cAMP-response Element-binding Protein and p110β Isoform of Phosphatidylinositol 3-Kinase. J. Biol. Chem..

[60] Yusni Y., Yusuf H., Murzalina C., Mahmudati N. (2026). Synergistic Modulation of Lipid Levels by Coffee and Swimming with Evidence of a Strong Obesity–Dyslipidemia Link: A Preclinical Study. Int. J. Food Sci..

[61] Edholm T., Degerblad M., Grybäck P., Hilsted L., Holst J.J., Jacobsson H., Efendic S., Schmidt P.T., Hellström P.M. (2010). Differential incretin effects of GIP and GLP-1 on gastric emptying, appetite, and insulin-glucose homeostasis. Neurogastroenterol. Motil..

[62] Xie X., Yang S., Deng S., Liu Y., Xu Z., He B. (2025). Comparative gastrointestinal adverse effects of GLP-1 receptor agonists and multi-target analogs in type 2 diabetes: A Bayesian network meta-analysis. Front. Pharmacol..

[63] Min T., Bain S.C. (2021). The Role of Tirzepatide, Dual GIP and GLP-1 Receptor Agonist, in the Management of Type 2 Diabetes: The SURPASS Clinical Trials. Diabetes Ther..

[64] Barana L., De Fano M., Cavallo M., Manco M., Prete D., Fanelli C.G., Porcellati F., Pippi R. (2025). Nutrition and Physical Activity in Optimizing Weight Loss and Lean Mass Preservation in the Incretin-Based Medications Era: A Narrative Review. Nutrients.

[65] Karl J.P., Roberts S.B. (2014). Energy Density, Energy Intake, and Body Weight Regulation in Adults. Adv. Nutr. Int. Rev. J..

[66] Nicklas B.J., Chmelo E., Delbono O., Carr J.J., Lyles M.F., Marsh A.P. (2015). Effects of resistance training with and without caloric restriction on physical function and mobility in overweight and obese older adults: A randomized controlled trial. Am. J. Clin. Nutr..

[67] Cava E., Yeat N.C., Mittendorfer B. (2017). Preserving Healthy Muscle during Weight Loss. Adv. Nutr. Int. Rev. J..

[68] Jacob E., Moura A., Avery A. (2024). A systematic review of physical activity and nutritional interventions for the management of normal weight and overweight obesity. Nutr. Metab. Cardiovasc. Dis..

[69] Çelik Ö., Yıldız B.O. (2021). Obesity and physical exercise. Minerva Endocrinol..

[70] Fitch A., Gigliotti L., Bays H.E. (2025). Application of nutrition interventions with GLP-1 based therapies: A narrative review of the challenges and solutions. Obes. Pillars.

[71] Miller G.D. (2019). Appetite Regulation: Hormones, Peptides, and Neurotransmitters and Their Role in Obesity. Am. J. Lifestyle Med..

[72] Hoffmann L., Egert S., Allgaier J., Kohlenberg-Müller K. (2023). Review of Validated Methods to Evaluate Diet History in Diet Therapy and Counselling: An Overview and Analysis of Screeners Based on Food-Based Dietary Guidelines. Nutrients.

[73] Hauser M.E., Hartle J.C., Landry M.J., Fielding-Singh P., Shih C.W., Qin F., Rigdon J., Gardner C.D. (2024). Association of dietary adherence and dietary quality with weight loss success among those following low-carbohydrate and low-fat diets: A secondary analysis of the DIETFITS randomized clinical trial. Am. J. Clin. Nutr..

[74] Simancas-Racines D., Frias-Toral E., Campuzano-Donoso M., Ramos-Sarmiento D., Zambrano-Villacres R., Reytor-González C., Schiavo L. (2025). Preoperative Nutrition in Bariatric Surgery: A Narrative Review on Enhancing Surgical Success and Patient Outcomes. Nutrients.

[75] Reytor-González C., Simancas-Racines D., Campuzano-Donoso M., Jimenez J.C., Román-Galeano N.M., Sarno G., Frias-Toral E. (2025). Harnessing nutrition to combat MASLD: A comprehensive guide to food-based therapeutic strategies. Food Agric. Immunol..

[76] Thomsen R.W., Mailhac A., Løhde J.B., Pottegård A. (2025). Real-world evidence on the utilization, clinical and comparative effectiveness, and adverse effects of newer GLP-1RA-based weight-loss therapies. Diabetes Obes. Metab..

[77] Dagan S.S., Goldenshluger A., Globus I., Schweiger C., Kessler Y., Sandbank G.K., Ben-Porat T., Sinai T. (2017). Nutritional Recommendations for Adult Bariatric Surgery Patients: Clinical Practice. Adv. Nutr. Int. Rev. J..

[78] Ben-Porat T., Sherf-Dagan S., Côté M., Miner C.J., Buch A. (2025). Nutritional Challenges of Incretin-Based Obesity Management Medications: Implications for Clinical Practice. Adv. Nutr. Int. Rev. J..

[79] Blake M.R., Raker J.M., Whelan K. (2016). Validity and reliability of the Bristol Stool Form Scale in healthy adults and patients with diarrhoea-predominant irritable bowel syndrome. Aliment. Pharmacol. Ther..

[80] Revicki D.A., Wood M., Wiklund I., Crawley J. (1997). Reliability and validity of the gastrointestinal symptom rating scale in patients with gastroesophageal reflux disease. Qual. Life Res..

[81] Shankar V., Thompson K.H., Wylie-Rosett J., Segal-Isaacson C.J. (2023). Validation and reliability for the updated REAP-S dietary screener, (Rapid Eating Assessment of Participants, Short Version, v.2). BMC Nutr..

[82] Segal-Isaacson C.J., Wylie-Rosett J., Gans K.M. (2004). Validation of a Short Dietary Assessment Questionnaire: The Rapid Eating and Activity Assessment for Participants Short Version (REAP-S). Diabetes Educ..

[83] Al-Najim W., Raposo A., BinMowyna M.N., le Roux C.W. (2025). Unintended Consequences of Obesity Pharmacotherapy: A Nutritional Approach to Ensuring Better Patient Outcomes. Nutrients.

[84] Westbury L.D., Durdin R., Robinson S.M., Cooper C., Cooper R., Ward K.A. (2025). Diet quality from mid-life and body composition in older age: Findings from a British birth cohort. Br. J. Nutr..

[85] Muscogiuri G., Verde L., Sulu C., Katsiki N., Hassapidou M., Frias-Toral E., Cucalón G., Pazderska A., Yumuk V.D., Colao A. (2022). Mediterranean Diet and Obesity-related Disorders: What is the Evidence?. Curr. Obes. Rep..

[86] Reytor-González C., Zambrano A.K., Frias-Toral E., Campuzano-Donoso M., Simancas-Racines D. (2025). Mediterranean diet and breast cancer: A narrative review. Medwave.

[87] Yoon D.Y., Sunwoo J., Shin N., Kim A.R., Kim B.T., Song G.S., Jang I., Lee S. (2021). Effect of meal timing on pharmacokinetics and pharmacodynamics of tegoprazan in healthy male volunteers. Clin. Transl. Sci..

[88] Silva N., Gomes N., Teixeira B. (2026). Systematic review: Mediterranean diet adherence and health outcomes in children and adolescents with ADHD and OCTD. Mediterr. J. Nutr. Metab..

[89] Godos J., Micek A., Di Venuta C., Di Mauro A., Furnari F., Balzano R.M., Di Giorgio J., Leonardi A., Caruso G., Grosso G. (2025). Role of Mediterranean diet in the prevention of cognitive decline: Biological mechanisms behind longevity promotion. Mediterr. J. Nutr. Metab..

[90] Gigliotti L., Warshaw H., Evert A., Dawkins C., Schwartz J., Susie C., Kushner R., Subramanian S., Handu D., Rozga M. (2025). Incretin-Based Therapies and Lifestyle Interventions: The Evolving Role of Registered Dietitian Nutritionists in Obesity Care. J. Acad. Nutr. Diet..

[91] Tinsley G.M., Nadolsky S. (2025). Preservation of lean soft tissue during weight loss induced by GLP-1 and GLP-1/GIP receptor agonists: A case series. SAGE Open Med. Case Rep..

[92] Beasley J.M., Shikany J.M., Thomson C.A. (2013). The Role of Dietary Protein Intake in the Prevention of Sarcopenia of Aging. Nutr. Clin. Pract..

[93] Bopp M.J., Houston D.K., Lenchik L., Easter L., Kritchevsky S.B., Nicklas B.J. (2008). Lean Mass Loss Is Associated with Low Protein Intake during Dietary-Induced Weight Loss in Postmenopausal Women. J. Am. Diet. Assoc..

[94] Kerlikowsky F., Krämer K., Eggersdorfer M., Hahn A. (2025). GLP-1 Receptor Agonists—Good for Body Weight, Bad for Micronutrient Status?. Curr. Dev. Nutr..

[95] Spreckley M., Ruggiero C.F., Brown A. (2026). Bridging the nutrition guidance gap for GLP-1 receptor agonist therapy assisted weight loss: Lessons from bariatric surgery. Int. J. Obes..

[96] Rubino D.M., Pedersen S.D., Connery L., Cao D., Chigutsa F., Stefanski A., Brumm J.F., Griffin R., Gerber C. (2025). Gastrointestinal tolerability and weight reduction associated with tirzepatide in adults with obesity or overweight with and without type 2 diabetes in the SURMOUNT-1 to -4 trials. Diabetes Obes. Metab..

[97] Ghusn W., Hurtado M.D. (2024). Glucagon-like Receptor-1 agonists for obesity: Weight loss outcomes, tolerability, side effects, and risks. Obes. Pillars.

[98] Bellini M., Tonarelli S., Barracca F., Rettura F., Pancetti A., Ceccarelli L., Ricchiuti A., Costa F., de Bortoli N., Marchi S. (2021). Chronic Constipation: Is a Nutritional Approach Reasonable?. Nutrients.

[99] Rollet M., Bohn T., Vahid F., on behalf of the ORISCAV Working Group (2021). Association between Dietary Factors and Constipation in Adults Living in Luxembourg and Taking Part in the ORISCAV-LUX 2 Survey. Nutrients.

[100] Valitova E.R., Bayrakci B., Bor S. (2013). The effect of the speed of eating on acid reflux and symptoms of patients with gastroesophageal reflux disease. Turk. J. Gastroenterol..

[101] Kang J.-E., Kang J. (2015). Lifestyle measures in the management of gastro-oesophageal reflux disease: Clinical and pathophysiological considerations. Ther. Adv. Chronic Dis..

[102] Ismaiel A., Scarlata G.G.M., Boitos I., Leucuta D.-C., Popa S.-L., Al Srouji N., Abenavoli L., Dumitrascu D.L. (2025). Gastrointestinal adverse events associated with GLP-1 RA in non-diabetic patients with overweight or obesity: A systematic review and network meta-analysis. Int. J. Obes..

[103] Atlantis E., Wu R., Dixon J. (2026). Incretin-based therapies for obesity and disordered eating: Optimising care in general practice. Fam. Med. Community Health.

[104] Aoun L., Almardini S., Saliba F., Haddadin F., Mourad O., Jdaidani J., Morcos Z., Al Saidi I., Sanayeh E.B., Saliba S. (2024). GLP-1 receptor agonists: A novel pharmacotherapy for binge eating (Binge eating disorder and bulimia nervosa)? A systematic review. J. Clin. Transl. Endocrinol..

[105] Bartel S., McElroy S.L., Levangie D., Keshen A. (2024). Use of glucagon-like peptide-1 receptor agonists in eating disorder populations. Int. J. Eat. Disord..

[106] Lister N.B., Baur L.A., Paxton S.J., Garnett S.P., Ahern A.L., Wilfley D.E., Maguire S., Sainsbury A., Steinbeck K., Braet C. (2024). Eating Disorders In weight-related Therapy (EDIT) Collaboration: Rationale and study design. Nutr. Res. Rev..

[107] Marucci S., Busetto L., Chianelli M., Fusco A., Carpentieri M., Armellini M., Tassone F., Sciaraffia M., Ponziani M.C., Nelva A. (2024). Screening, Diagnosis, and Treatment of Patients with Binge Eating Disorder and Obesity: What the Endocrinologist Needs to Know. Endocrines.

[108] Krabbenborg M.A.M., Danner U.N., Larsen J.K., van der Veer N., van Elburg A.A., de Ridder D.T.D., Evers C., Stice E., Engels R.C.M.E. (2012). The Eating Disorder Diagnostic Scale: Psychometric Features Within a Clinical Population and a Cut-off Point to Differentiate Clinical Patients from Healthy Controls. Eur. Eat. Disord. Rev..

[109] Morgan J.F., Reid F., Lacey J.H. (2000). The SCOFF questionnaire: A new screening tool for eating disorders. West. J. Med..

[110] Solmi F., Hatch S.L., Hotopf M., Treasure J., Micali N. (2015). Validation of the SCOFF questionnaire for eating disorders in a multiethnic general population sample. Int. J. Eat. Disord..

[111] Espel-Huynh H., Thompson-Brenner H., Boswell J.F., Zhang F., Juarascio A.S., Lowe M.R. (2020). Development and validation of a progress monitoring tool tailored for use in intensive eating disorder treatment. Eur. Eat. Disord. Rev..

[112] Freiberger E., Goisser S., Porzel S., Volkert D., Kemmler W., Sieber C., Bollheimer C. (2015). Sarcopenic obesity and complex interventions with nutrition and exercise in community-dwelling older persons—A narrative review. Clin. Interv. Aging.

[113] Morley J.E., Anker S.D., von Haehling S. (2014). Prevalence, incidence, and clinical impact of sarcopenia: Facts, numbers, and epidemiology—Update 2014. J. Cachexia Sarcopenia Muscle.

[114] Villareal D.T., Aguirre L., Gurney A.B., Waters D.L., Sinacore D.R., Colombo E., Armamento-Villareal R., Qualls C. (2017). Aerobic or Resistance Exercise, or Both, in Dieting Obese Older Adults. N. Engl. J. Med..

[115] Fuchs C.J., van Loon L.J. (2025). Muscle preservation during hospitalization: Energy balance, protein intake, and habitual physical activity. Curr. Opin. Clin. Nutr. Metab. Care.

[116] Bruyere O., Beaudart C., Reginster J.-Y., Buckinx F., Schoene D., Hirani V., Cooper C., Kanis J.A., Rizzoli R., McCloskey E. (2016). Assessment of muscle mass, muscle strength and physical performance in clinical practice: An international survey. Eur. Geriatr. Med..

[117] Cruz-Jentoft A.J., Bahat G., Bauer J., Boirie Y., Bruyère O., Cederholm T., Cooper C., Landi F., Rolland Y., Sayer A.A. (2019). Sarcopenia: Revised European consensus on definition and diagnosis. Age Ageing.

[118] Cohen R., Fernie G., Fekr A.R. (2021). Fluid Intake Monitoring Systems for the Elderly: A Review of the Literature. Nutrients.

[119] Chen X., Kamavuako E.N. (2023). Vision-Based Methods for Food and Fluid Intake Monitoring: A Literature Review. Sensors.

[120] Frias-Toral E., Chapela S., Gonzalez V., Martinuzzi A., Locatelli J., Llobera N., Manrique E., Sarno G., Mingo M., Marchese F. (2025). Optimizing Nutritional Management Before and After Bariatric Surgery: A Comprehensive Guide for Sustained Weight Loss and Metabolic Health. Nutrients.

[121] Wharton S., Calanna S., Davies M., Dicker D., Goldman B., Lingvay I., Mosenzon O., Rubino D.M., Thomsen M., Wadden T.A. (2022). Gastrointestinal tolerability of once-weekly semaglutide 2.4 mg in adults with overweight or obesity, and the relationship between gastrointestinal adverse events and weight loss. Diabetes Obes. Metab..

[122] Laudisio D., Muscogiuri G., Barrea L., Savastano S., Colao A. (2018). Obesity and breast cancer in premenopausal women: Current evidence and future perspectives. Eur. J. Obstet. Gynecol. Reprod. Biol..

[123] Cifuentes L., Camilleri M., Acosta A. (2021). Gastric Sensory and Motor Functions and Energy Intake in Health and Obesity—Therapeutic Implications. Nutrients.

[124] Feinle-Bisset C., Azpiroz F. (2013). Dietary Lipids and Functional Gastrointestinal Disorders. Am. J. Gastroenterol..

[125] Manne-Goehler J., Franco J. (2025). Side effects of GLP-1 receptor agonists. BMJ.

[126] van Nieuwenhoven M.A., Vriens B.E.P.J., Brummer R.-J.M., Brouns F. (2000). Effect of dehydration on gastrointestinal function at rest and during exercise in humans. Eur. J. Appl. Physiol..

[127] Liska D., Mah E., Brisbois T., Barrios P.L., Baker L.B., Spriet L.L. (2019). Narrative Review of Hydration and Selected Health Outcomes in the General Population. Nutrients.

[128] Trapanese V., Dagostino A., Natale M.R., Giofrè F., Vatalaro C., Melina M., Cosentino F., Sergi S., Imoletti F., Spagnuolo R. (2025). Bidirectional Interactions Between the Gut Microbiota and Incretin-Based Therapies. Life.

[129] Garvey W.T., Batterham R.L., Bhatta M., Buscemi S., Christensen L.N., Frias J.P., Jódar E., Kandler K., Rigas G., Wadden T.A. (2022). Two-year effects of semaglutide in adults with overweight or obesity: The STEP 5 trial. Nat. Med..

[130] van der Schoot A., Drysdale C., Whelan K., Dimidi E. (2022). The Effect of Fiber Supplementation on Chronic Constipation in Adults: An Updated Systematic Review and Meta-Analysis of Randomized Controlled Trials. Am. J. Clin. Nutr..

[131] Dahl W.J., Stewart M.L. (2015). Position of the Academy of Nutrition and Dietetics: Health Implications of Dietary Fiber. J. Acad. Nutr. Diet..

[132] McRorie J.W. (2015). Evidence-Based Approach to Fiber Supplements and Clinically Meaningful Health Benefits, Part 2. Nutr. Today.

[133] Rao S.S., Brenner D.M. (2021). Efficacy and Safety of Over-the-Counter Therapies for Chronic Constipation: An Updated Systematic Review. Am. J. Gastroenterol..

[134] Wan J., Ferrari C., Tadros M. (2024). GLP-1RA Essentials in Gastroenterology: Side Effect Management, Precautions for Endoscopy and Applications for Gastrointestinal Disease Treatment. Gastroenterol. Insights.

[135] Amerikanou C., Kleftaki S.-A., Valsamidou E., Chroni E., Biagki T., Sigala D., Koutoulogenis K., Anapliotis P., Gioxari A., Kaliora A.C. (2023). Food, Dietary Patterns, or Is Eating Behavior to Blame? Analyzing the Nutritional Aspects of Functional Dyspepsia. Nutrients.

[136] Mäkinen K.K. (2016). Gastrointestinal Disturbances Associated with the Consumption of Sugar Alcohols with Special Consideration of Xylitol: Scientific Review and Instructions for Dentists and Other Health-Care Professionals. Int. J. Dent..

[137] Wilding J.P.H., Batterham R.L., Davies M., Van Gaal L.F., Kandler K., Konakli K., Lingvay I., McGowan B.M., Oral T.K., Rosenstock J. (2022). Weight regain and cardiometabolic effects after withdrawal of semaglutide: The STEP 1 trial extension. Diabetes Obes. Metab..

[138] Heymsfield S.B., Gonzalez M.C.C., Shen W., Redman L., Thomas D. (2014). Weight loss composition is one-fourth fat-free mass: A critical review and critique of this widely cited rule. Obes. Rev..

[139] Look M., Dunn J.P., Kushner R.F., Cao D., Harris C., Gibble T.H., Stefanski A., Griffin R. (2025). Body composition changes during weight reduction with tirzepatide in the SURMOUNT-1 study of adults with obesity or overweight. Diabetes Obes. Metab..

[140] Hall K.D., Kahan S. (2018). Maintenance of Lost Weight and Long-Term Management of Obesity. Med. Clin. N. Am..

[141] American Diabetes Association Professional Practice Committee (2024). 8. Obesity and Weight Management for the Prevention and Treatment of Type 2 Diabetes: Standards of Care in Diabetes–2025. Diabetes Care.

[142] Kim J.Y. (2021). Optimal Diet Strategies for Weight Loss and Weight Loss Maintenance. J. Obes. Metab. Syndr..

[143] Haaf D.S.T., Eijsvogels T.M., Bongers C.C., Horstman A.M., Timmers S., de Groot L.C., Hopman M.T. (2019). Protein supplementation improves lean body mass in physically active older adults: A randomized placebo-controlled trial. J. Cachexia Sarcopenia Muscle.

[144] Nunes E.A., Colenso-Semple L., McKellar S.R., Yau T., Ali M.U., Fitzpatrick-Lewis D., Sherifali D., Gaudichon C., Tomé D., Atherton P.J. (2022). Systematic review and meta-analysis of protein intake to support muscle mass and function in healthy adults. J. Cachexia Sarcopenia Muscle.

[145] Schiavo L., Santella B., Paolini B., Rahimi F., Giglio E., Martinelli B., Boschetti S., Bertolani L., Gennai K., Arolfo S. (2024). Adding Branched-Chain Amino Acids and Vitamin D to Whey Protein Is More Effective than Protein Alone in Preserving Fat Free Mass and Muscle Strength in the First Month after Sleeve Gastrectomy. Nutrients.

[146] Leidy H.J., Clifton P.M., Astrup A., Wycherley T.P., Westerterp-Plantenga M.S., Luscombe-Marsh N.D., Woods S.C., Mattes R.D. (2015). The role of protein in weight loss and maintenance. Am. J. Clin. Nutr..

[147] Kim J.E., O’connor L.E., Sands L.P., Slebodnik M.B., Campbell W.W. (2016). Effects of dietary protein intake on body composition changes after weight loss in older adults: A systematic review and meta-analysis. Nutr. Rev..

[148] Bauer J., Biolo G., Cederholm T., Cesari M., Cruz-Jentoft A.J., Morley J.E., Phillips S., Sieber C., Stehle P., Teta D. (2013). Evidence-Based Recommendations for Optimal Dietary Protein Intake in Older People: A Position Paper From the PROT-AGE Study Group. J. Am. Med. Dir. Assoc..

[149] Donini L.M., Poggiogalle E., Migliaccio S., Aversa A., Pinto A. (2013). Body composition in sarcopenic obesity: Systematic review of the literature. Mediterr. J. Nutr. Metab..

[150] Prokopidis K., Daly R.M., Suetta C. (2025). Weighing the risk of GLP-1 treatment in older adults: Should we be concerned about sarcopenic obesity?. J. Nutr. Health Aging.

[151] Schoenfeld B.J., Aragon A.A. (2018). How much protein can the body use in a single meal for muscle-building? Implications for daily protein distribution. J. Int. Soc. Sports Nutr..

[152] Paddon-Jones D., Rasmussen B.B. (2009). Dietary protein recommendations and the prevention of sarcopenia. Curr. Opin. Clin. Nutr. Metab. Care.

[153] Layman D.K. (2024). Impacts of protein quantity and distribution on body composition. Front. Nutr..

[154] Rondanelli M., Nichetti M., Peroni G., Faliva M.A., Naso M., Gasparri C., Perna S., Oberto L., Di Paolo E., Riva A. (2021). Where to Find Leucine in Food and How to Feed Elderly With Sarcopenia in Order to Counteract Loss of Muscle Mass: Practical Advice. Front. Nutr..

[155] Massimino E., Izzo A., Castaldo C., Amoroso A.P., Rivellese A.A., Capaldo B., Della Pepa G. (2023). Protein and Leucine Intake at Main Meals in Elderly People with Type 2 Diabetes. Nutrients.

[156] Volek J.S., Kackley M.L., Buga A. (2024). Nutritional Considerations During Major Weight Loss Therapy: Focus on Optimal Protein and a Low-Carbohydrate Dietary Pattern. Curr. Nutr. Rep..

[157] Van Vliet S., Beals J.W., Martinez I.G., Skinner S.K., Burd N.A. (2018). Achieving Optimal Post-Exercise Muscle Protein Remodeling in Physically Active Adults through Whole Food Consumption. Nutrients.

[158] Moon J., Koh G. (2020). Clinical Evidence and Mechanisms of High-Protein Diet-Induced Weight Loss. J. Obes. Metab. Syndr..

[159] Pasiakos S.M., Cao J.J., Margolis L.M., Sauter E.R., Whigham L.D., McClung J.P., Rood J.C., Carbone J.W., Combs G.F., Young A.J. (2013). Effects of high-protein diets on fat-free mass and muscle protein synthesis following weight loss: A randomized controlled trial. FASEB J..

[160] Morton R.W., Murphy K.T., McKellar S.R., Schoenfeld B.J., Henselmans M., Helms E., Aragon A.A., Devries M.C., Banfield L., Krieger J.W. (2018). A systematic review, meta-analysis and meta-regression of the effect of protein supplementation on resistance training-induced gains in muscle mass and strength in healthy adults. Br. J. Sports Med..

[161] Esmarck B., Andersen J.L., Olsen S., Richter E.A., Mizuno M., Kjær M. (2001). Timing of postexercise protein intake is important for muscle hypertrophy with resistance training in elderly humans. J. Physiol..

[162] Jäger R., Kerksick C.M., Campbell B.I., Cribb P.J., Wells S.D., Skwiat T.M., Purpura M., Ziegenfuss T.N., Ferrando A.A., Arent S.M. (2017). International Society of Sports Nutrition Position Stand: Protein and exercise. J. Int. Soc. Sports Nutr..

[163] Lowe D.A., Wu N., Rohdin-Bibby L., Moore A.H., Kelly N., Liu Y.E., Philip E., Vittinghoff E., Heymsfield S.B., Olgin J.E. (2020). Effects of Time-Restricted Eating on Weight Loss and Other Metabolic Parameters in Women and Men With Overweight and Obesity. JAMA Intern. Med..

[164] Mesinovic J., Hurst C., Leung G.K.W., Ryan J.R., Daly R.M., Scott D. (2025). Exercise and dietary recommendations to preserve musculoskeletal health during weight loss in adults with obesity: A practical guide. Rev. Endocr. Metab. Disord..

[165] Miller T., Mull S., Aragon A.A., Krieger J., Schoenfeld B.J. (2018). Resistance Training Combined With Diet Decreases Body Fat While Preserving Lean Mass Independent of Resting Metabolic Rate: A Randomized Trial. Int. J. Sport Nutr. Exerc. Metab..

[166] Oliveira G.S., Vieira F.T., Lamarca F., Lima R.M., Carvalho K.M.B., Dutra E.S. (2021). Resistance Training Improves Muscle Strength and Function, Regardless of Protein Supplementation, in the Mid- to Long-Term Period after Gastric Bypass. Nutrients.

[167] Whaikid P., Piaseu N. (2024). The effectiveness of protein supplementation combined with resistance exercise programs among community-dwelling older adults with sarcopenia: A systematic review and meta-analysis. Epidemiol. Health.

[168] Binmahfoz A., Dighriri A., Gray C., Gray S.R. (2025). Effect of resistance exercise on body composition, muscle strength and cardiometabolic health during dietary weight loss in people living with overweight or obesity: A systematic review and meta-analysis. BMJ Open Sport Exerc. Med..

[169] Huiberts R.O., Wüst R.C.I., van der Zwaard S. (2024). Concurrent Strength and Endurance Training: A Systematic Review and Meta-Analysis on the Impact of Sex and Training Status. Sports Med..

[170] Lundberg T.R., Feuerbacher J.F., Sünkeler M., Schumann M. (2022). The Effects of Concurrent Aerobic and Strength Training on Muscle Fiber Hypertrophy: A Systematic Review and Meta-Analysis. Sports Med..

[171] Brown J., Clarke C., Johnson S.C., Sievenpiper J. (2022). Canadian Adult Obesity Clinical Practice Guidelines: Medical Nutrition Therapy in Obesity Management. https://obesitycanada.ca/guidelines/nutrition.

[172] Smethers A.D., Rolls B.J. (2018). Dietary Management of Obesity. Med. Clin. N. Am..

[173] Godos J., Guglielmetti M., Ferraris C., Frias-Toral E., Azpíroz I.D., Lipari V., Di Mauro A., Furnari F., Castellano S., Galvano F. (2025). Mediterranean Diet and Quality of Life in Adults: A Systematic Review. Nutrients.

[174] Barrea L., Muscogiuri G., Frias-Toral E., Laudisio D., Pugliese G., Castellucci B., Garcia-Velasquez E., Savastano S., Colao A. (2021). Nutrition and immune system: From the Mediterranean diet to dietary supplementary through the microbiota. Crit. Rev. Food Sci. Nutr..

[175] Barrea L., Pugliese G., Laudisio D., Colao A., Savastano S., Muscogiuri G. (2021). Mediterranean diet as medical prescription in menopausal women with obesity: A practical guide for nutritionists. Crit. Rev. Food Sci. Nutr..

[176] Monaco A., Verde L., Pignatelli M.F., Docimo A., Ferrandes S., Barrea L., Calisti F., Cozzolino G., Muscogiuri G., Docimo G. (2025). Adherence to Mediterranean diet and prevalence of differentiated thyroid cancer: A single-center Unit of Thyroid Surgery experience in a Southern-Italy cohort. Minerva Endocrinol..

[177] Savanelli M.C., Barrea L., Macchia P.E., Savastano S., Falco A., Renzullo A., Scarano E., Nettore I.C., Colao A., Di Somma C. (2017). Preliminary results demonstrating the impact of Mediterranean diet on bone health. J. Transl. Med..

[178] Di Mauro A., Tuccinardi D., Watanabe M., Del Toro R., Monte L., Giorgino R., Rampa L., Rossini G., Kyanvash S., Soare A. (2021). The Mediterranean diet increases glucagon-like peptide 1 and oxyntomodulin compared with a vegetarian diet in patients with type 2 diabetes: A randomized controlled cross-over trial. Diabetes/Metab. Res. Rev..

[179] Barrea L., Tarantino G., Di Somma C., Muscogiuri G., Macchia P.E., Falco A., Colao A., Savastano S. (2017). Adherence to the Mediterranean Diet and Circulating Levels of Sirtuin 4 in Obese Patients: A Novel Association. Oxid. Med. Cell. Longev..

[180] Tosti V., Bertozzi B., Fontana L. (2018). Health Benefits of the Mediterranean Diet: Metabolic and Molecular Mechanisms. J. Gerontol. Ser. A.

[181] Tolomeo M., De Carli L., Guidi S., Zanardi M., Giacomini D., Devecchi C., Pistone E., Ponta M., Simonetti P., Sykes K. (2023). The Mediterranean Diet: From the pyramid to the circular model. Mediterr. J. Nutr. Metab..

[182] Gofron K.K., Wasilewski A., Małgorzewicz S. (2025). Effects of GLP-1 Analogues and Agonists on the Gut Microbiota: A Systematic Review. Nutrients.

[183] Kamath S., Chan N.S.L., Joyce P. (2026). GLP-1 agonists and the gut microbiome: A bidirectional relationship. Br. J. Clin. Pharmacol..

[184] Sato J., Kanazawa A., Makita S., Hatae C., Komiya K., Shimizu T., Ikeda F., Tamura Y., Ogihara T., Mita T. (2017). A randomized controlled trial of 130 g/day low-carbohydrate diet in type 2 diabetes with poor glycemic control. Clin. Nutr..

[185] Ludwig D.S., Dickinson S.L., Henschel B., Ebbeling C.B., Allison D.B. (2021). Do Lower-Carbohydrate Diets Increase Total Energy Expenditure? An Updated and Reanalyzed Meta-Analysis of 29 Controlled-Feeding Studies. J. Nutr..

[186] Gomez-Arbelaez D., Crujeiras A.B., Castro A.I., Martinez-Olmos M.A., Canton A., Ordoñez-Mayan L., Sajoux I., Galban C., Bellido D., Casanueva F.F. (2018). Resting metabolic rate of obese patients under very low calorie ketogenic diet. Nutr. Metab..

[187] Reytor-González C., Simancas-Racines D., Román-Galeano N.M., Campuzano-Donoso M., Carella A.M., Zambrano-Villacres R., Marinelli T., Coppola L., Marchetti M., Galasso M. (2025). Obesity and breast cancer: Exploring the nexus of chronic inflammation, metabolic dysregulation, and nutritional strategies. Food Agric. Immunol..

[188] Muscogiuri G., Verde L., Frias-Toral E., Reytor-González C., Annunziata G., Proganò M., Savastano S., Simancas-Racines D., Colao A., Barrea L. (2024). Weight loss, changes in body composition and inflammatory status after a very low-energy ketogenic therapy (VLEKT): Does gender matter?. J. Transl. Med..

[189] Pilone V., Tramontano S., Renzulli M., Romano M., Cobellis L., Berselli T., Schiavo L. (2018). Metabolic effects, safety, and acceptability of very low-calorie ketogenic dietetic scheme on candidates for bariatric surgery. Surg. Obes. Relat. Dis..

[190] Schiavo L., Santella B., Mingo M., Rossetti G., Orio M., Cobellis L., Maurano A., Iannelli A., Pilone V. (2025). Preliminary Evidence Suggests That a 12-Week Treatment with Tirzepatide Plus Low-Energy Ketogenic Therapy Is More Effective than Its Combination with a Low-Calorie Diet in Preserving Fat-Free Mass, Muscle Strength, and Resting Metabolic Rate in Patients with Obesity. Nutrients.

[191] Bai M.R., Abirami K., Gayathri R., Vedantham S., Shobana S., Nagarajan L.P., Gunasekaran G., Nagamuthu G., Malini H.M., Gokulakrishnan K. (2024). Effect of low vs. high dietary-advanced glycation end products on insulin-sensitivity and inflammatory- markers among overweight/obese Asian-Indian adults-A randomised controlled trial. Int. J. Food Sci. Nutr..

[192] Karras S.N., Koufakis T., Dimakopoulos G., Popovic D.S., Adamidou L., Makedou K., Kotsa K. (2024). The Mediterranean diet, but not time-restricted eating, mediates the effects of nesfatin on beta cell function among overweight, metabolically healthy individuals. Int. J. Food Sci. Nutr..

[193] Kooij K.L., Koster D.I., Eeltink E., Luijendijk M., Drost L., Ducrocq F., Adan R.A. (2024). GLP-1 receptor agonist semaglutide reduces appetite while increasing dopamine reward signaling. Neurosci. Appl..

[194] Caprara G. (2018). Diet and longevity: The effects of traditional eating habits on human lifespan extension. Mediterr. J. Nutr. Metab..

[195] Koide Y., Kato T., Hayashi M., Daido H., Maruyama T., Ishihara T., Nishimura K., Tsunekawa S., Yabe D. (2025). Association between eating behavior patterns and the therapeutic efficacy of GLP-1 receptor agonists in individuals with type 2 diabetes: A multicenter prospective observational study. Front. Clin. Diabetes Health.

[196] Fitzpatrick S.L., Appel L.J., Bray B., Brooks N., Stevens V.J. (2018). Predictors of Long-Term Adherence to Multiple Health Behavior Recommendations for Weight Management. Health Educ. Behav..

[197] Karfopoulou E., Brikou D., Mamalaki E., Bersimis F., Anastasiou C.A., Hill J.O., Yannakoulia M. (2017). Dietary patterns in weight loss maintenance: Results from the MedWeight study. Eur. J. Nutr..

[198] Suresh A., Shobna, Salaria M., Morya S., Khalid W., Afzal F.A., Khan A.A., Safdar S., Khalid M.Z., Kasongo E.L.M. (2024). Dietary fiber: An unmatched food component for sustainable health. Food Agric. Immunol..

[199] von Muhlenbrock C., Aronsohn F., Quera R., Madrid A.M. (2025). The role of dietary fiber in the gastrointestinal tract: When, how and why?. Best Pract. Res. Clin. Gastroenterol..

[200] Reytor-González C., Zambrano A.K., Montalvan M., Frias-Toral E., Simancas-Racines A., Simancas-Racines D. (2024). Adherence to the Mediterranean Diet and its association with gastric cancer: Health benefits from a Planeterranean perspective. J. Transl. Med..

[201] Scaglione S., Di Chiara T., Daidone M., Tuttolomondo A. (2025). Effects of the Mediterranean Diet on the Components of Metabolic Syndrome Concerning the Cardiometabolic Risk. Nutrients.

[202] Locatelli J.C., Costa J.G., Haynes A., Naylor L.H., Fegan P.G., Yeap B.B., Green D.J. (2024). Incretin-Based Weight Loss Pharmacotherapy: Can Resistance Exercise Optimize Changes in Body Composition?. Diabetes Care.

[203] Gualano B., Kirwan J.P., Roschel H. (2021). Exercise Is Key to Sustaining Metabolic Gains After Bariatric Surgery. Exerc. Sport Sci. Rev..

[204] Harris S., DePalma J., Barkoukis H. (2025). Protein and Aging: Practicalities and Practice. Nutrients.

[205] Ikizler T.A., Burrowes J.D., Byham-Gray L.D., Campbell K.L., Carrero J.-J., Chan W., Fouque D., Friedman A.N., Ghaddar S., Goldstein-Fuchs D.J. (2020). KDOQI Clinical Practice Guideline for Nutrition in CKD: 2020 Update. Am. J. Kidney Dis..

[206] Ko G.J., Obi Y., Tortorici A.R., Kalantar-Zadeh K. (2017). Dietary protein intake and chronic kidney disease. Curr. Opin. Clin. Nutr. Metab. Care.

[207] Aronne L.J., Sattar N., Horn D.B., Bays H.E., Wharton S., Lin W.-Y., Ahmad N.N., Zhang S., Liao R., Bunck M.C. (2024). Continued Treatment With Tirzepatide for Maintenance of Weight Reduction in Adults With Obesity. JAMA.

[208] Brufani C., Morviducci L. (2025). Semaglutide in a real-world outpatient setting: Discontinuation patterns and weight maintenance. Obes. Endocrinol..

[209] Wu C.C., Cengiz A., Lawley S.D. (2025). Less frequent dosing of GLP-1 receptor agonists as a viable weight maintenance strategy. Obesity.

[210] Cengiz A., Wu C.C., Lawley S.D. (2025). Alternative dosing regimens of GLP-1 receptor agonists may reduce costs and maintain weight loss efficacy. Diabetes Obes. Metab..

[211] Holt J., Sandsdal R.M., Byberg S., Janus C., Juhl C.R., Jørgensen J.R., Hartmann B., Stallknecht B., Holst J.J., Madsbad S. (2026). One Year of Exercise After Weight Loss Increases Postprandial GLP-1 Secretion in Contrast to Usual Activity or GLP-1 Receptor Agonist Treatment. Obesity.

[212] Annunziata G., Paoli A., Manzi V., Camajani E., Laterza F., Verde L., Capó X., Padua E., Bianco A., Carraro A. (2024). The Role of Physical Exercise as a Therapeutic Tool to Improve Lipedema: A Consensus Statement from the Italian Society of Motor and Sports Sciences (Società Italiana di Scienze Motorie e Sportive, SISMeS) and the Italian Society of Phlebology (Società Italiana di Flebologia, SIF). Curr. Obes. Rep..

[213] Yang Y., He L., Han S., Yang N., Liu Y., Wang X., Li Z., Ping F., Xu L., Li W. (2025). Sex Differences in the Efficacy of Glucagon-Like Peptide-1 Receptor Agonists for Weight Reduction: A Systematic Review and Meta-Analysis. J. Diabetes.

[214] Rentzeperi E., Pegiou S., Koufakis T., Grammatiki M., Kotsa K. (2022). Sex Differences in Response to Treatment with Glucagon-like Peptide 1 Receptor Agonists: Opportunities for a Tailored Approach to Diabetes and Obesity Care. J. Pers. Med..

[215] Millward D.J., Truby H., Fox K.R., Livingstone M.B.E., Macdonald I.A., Tothill P. (2013). Sex differences in the composition of weight gain and loss in overweight and obese adults. Br. J. Nutr..

[216] MacIsaac R.J., Trevella P., Ekinci E.I. (2024). Glucagon-like peptide-1 receptor agonists and kidney outcomes. J. Diabetes.

[217] Meier M., Cummings J., Abdelsaid M., Feliciano J., Eusebe J., Stirn H., Coucha M. (2026). Glucagon-Like Peptide-1 Receptor Agonists in Chronic Kidney Disease: Mechanisms and Clinical Perspectives. Kidney Med..

[218] Chen J.-Y., Hsu T.-W., Liu J.-H., Pan H.-C., Lai C.-F., Yang S.-Y., Wu V.-C. (2025). Kidney and Cardiovascular Outcomes Among Patients with CKD Receiving GLP-1 Receptor Agonists: A Systematic Review and Meta-Analysis of Randomized Trials. Am. J. Kidney Dis..

[219] De la Flor J.C., Lorenzo J.D., Marschall A., Valga F., Vázquez T.M., Cícero E.R. (2022). Efficacy and Safety of Semaglutide, a Glucagon-Like Peptide-1 Receptor Agonist in Real-Life: A Case Series of Patients in Maintenance Incremental Hemodialysis. Case Rep. Nephrol. Dial..

[220] Begum F., Chang K., Kapoor K., Vij R., Phadke G., Hiser W.M., Wanchoo R., Sharma P., Sutaria N., Jhaveri K.D. (2024). Semaglutide-associated kidney injury. Clin. Kidney J..

[221] Filippatos T.D., Panagiotopoulou T.V., Elisaf M.S. (2014). Adverse Effects of GLP-1 Receptor Agonists. Rev. Diabet. Stud..

[222] Schiavo L., Santella B., Mingo M., Rossetti G., Orio M., Pilone V. (2025). Beyond Weight Loss: Comparative Effects of Tirzepatide Plus Low-Energy Ketogenic Versus Low-Calorie Diet on Hepatic Steatosis and Stiffness in MASLD. Nutrients.

[223] Loomba R., Hartman M.L., Lawitz E.J., Vuppalanchi R., Boursier J., Bugianesi E., Yoneda M., Behling C., Cummings O.W., Tang Y. (2024). Tirzepatide for Metabolic Dysfunction–Associated Steatohepatitis with Liver Fibrosis. N. Engl. J. Med..

[224] Newsome P.N., Buchholtz K., Cusi K., Linder M., Okanoue T., Ratziu V., Sanyal A.J., Sejling A.-S., Harrison S.A. (2021). A Placebo-Controlled Trial of Subcutaneous Semaglutide in Nonalcoholic Steatohepatitis. N. Engl. J. Med..

[225] Grosso G., Laudisio D., Frias-Toral E., Barrea L., Muscogiuri G., Savastano S., Colao A. (2022). Anti-Inflammatory Nutrients and Obesity-Associated Metabolic-Inflammation: State of the Art and Future Direction. Nutrients.

[226] Sheikh M.Y., Younus M.F., Shergill A., Hasan M.N. (2025). Diet and Lifestyle Interventions in Metabolic Dysfunction-Associated Fatty Liver Disease: A Comprehensive Review. Int. J. Mol. Sci..

[227] Reytor-González C., Annunziata G., Campuzano-Donoso M., Morales-López T., Basantes-Tituaña C., Fascì-Spurio F., Verde L., Muscogiuri G., Barrea L., Frias-Toral E. (2025). Endocrinologist’s crucial role in metabolic dysfunction-associated steatotic liver disease: A comprehensive review. Minerva Endocrinol..

[228] Barrea L., Muscogiuri G., Macchia P.E., Di Somma C., Falco A., Savanelli M.C., Colao A., Savastano S. (2017). Mediterranean Diet and Phase Angle in a Sample of Adult Population: Results of a Pilot Study. Nutrients.

[229] Foster D., Sanchez-Collins S., Cheskin L.J. (2017). Multidisciplinary Team–Based Obesity Treatment in Patients With Diabetes: Current Practices and the State of the Science. Diabetes Spectr..

[230] Savas O., Kaya A. (2026). The impact of the number and frequency of visits on weight loss success in patients attending the obesity outpatient clinic. Medicine.

[231] Marra M.V., Lilly C.L., Nelson K.R., Woofter D.R., Malone J. (2019). A Pilot Randomized Controlled Trial of a Telenutrition Weight Loss Intervention in Middle-Aged and Older Men with Multiple Risk Factors for Cardiovascular Disease. Nutrients.

[232] Alolayan R.A., Aldisi D.A., Hussain D.S., Alafif N., Abulmeaty M.M.A. (2024). The Efficacy of Telehealth Versus In-Person Management Delivery in Adult Patients with Obesity. Healthcare.

[233] Chiavarini M., Giacchetta I., Rosignoli P., Fabiani R. (2025). E-Health and M-Health in Obesity Management: A Systematic Review and Meta-Analysis of RCTs. Nutrients.

[234] Auster-Gussman L.A., Rikhy M., Lockwood K.G., Branch O.H., Graham S.A. (2023). The Effects of Providing a Connected Scale in an App-Based Digital Health Program: Cross-sectional Examination. JMIR mHealth uHealth.

[235] Przybyłowski A., Górski M., Gwioździk W., Polaniak R. (2025). Redefining Obesity: A Narrative Review of Diagnostic Evolution, Therapeutic Strategies and Psychosocial Determinants. Healthcare.

[236] Simancas-Racines D., Campuzano-Donoso M., Román-Galeano N.M., Zambrano-Villacres R., Memoli P., Verde L., Reytor-González C., Carbone L. (2025). Obesity and endometrial cancer: Biological mechanisms, nutritional strategies, and clinical perspectives. Food Agric. Immunol..

[237] Ufholz K., Werner J. (2023). The Efficacy of Mobile Applications for Weight Loss. Curr. Cardiovasc. Risk Rep..

[238] Ahn J.S., Lee H., Kim J., Park H., Kim D.W., Lee J.E. (2020). Use of a Smartphone App for Weight Loss Versus a Paper-Based Dietary Diary in Overweight Adults: Randomized Controlled Trial. JMIR mHealth uHealth.

